# Hydrophobic-cationic peptides modulate RNA polymerase ribozyme activity by accretion

**DOI:** 10.1038/s41467-022-30590-3

**Published:** 2022-06-03

**Authors:** Peiying Li, Philipp Holliger, Shunsuke Tagami

**Affiliations:** 1 RIKEN Center for Biosystems Dynamics Research, 1-7-22 Suehiro-cho, Tsurumi-ku, Yokohama 230-0045 Japan; 2 MRC Laboratory of Molecular Biology, Francis Crick Avenue, Cambridge Biomedical Campus, Cambridge, CB2 0QH UK

**Keywords:** Peptides, RNA, Origin of life

## Abstract

Accretion and the resulting increase in local concentration is a widespread mechanism in biology to enhance biomolecular functions (for example, in liquid-liquid demixing phases). Such macromolecular aggregation phases (e.g., coacervates, amyloids) may also have played a role in the origin of life. Here, we report that a hydrophobic-cationic RNA binding peptide selected by phage display (P43: AKKVWIIMGGS) forms insoluble amyloid-containing aggregates, which reversibly accrete RNA on their surfaces in an RNA-length and Mg^2+^-concentration dependent manner. The aggregates formed by P43 or its sequence-simplified version (K_2_V_6_: KKVVVVVV) inhibited RNA polymerase ribozyme (RPR) activity at 25 mM MgCl_2_, while enhancing it significantly at 400 mM MgCl_2_. Our work shows that such hydrophobic-cationic peptide aggregates can reversibly concentrate RNA and enhance the RPR activity, and suggests that they could have aided the emergence and evolution of longer and functional RNAs in the fluctuating environments of the prebiotic earth.

## Abstract (web summary)

Macromolecular aggregates may have played an important role in the origin of life. Here, the authors report hydrophobic-cationic peptides that form insoluble aggregates, which reversibly accrete RNA on their surfaces, and enhance RNA polymerization by a ribozyme.

## Introduction

Accretion and self-organization of biopolymers (e.g., peptides and RNA) into defined locations were important steps in the emergence of life. Indeed, compartmentalization or spatial confinement are seen as particularly relevant for the emergence of genetic self-replication and Darwinian evolution, as otherwise genetic kin selection cannot occur and a replication system becomes vulnerable to being overrun by replication parasites^1,2^. In all modern organisms, cell membranes composed of phospholipids and, to some extent, polypeptide scaffolds and demixing phases, maintain the spatial organization and integration of complex macromolecular systems. However, it remains unclear how such organizing principles emerged on the prebiotic earth and what primordial molecules/structures were able to drive the accumulation of functional biopolymers.

As in modern organisms, lipid membranes might have served as an organizing principle and scaffold to enhance biomolecular interactions. As the synthesis of phospholipids under prebiotic conditions appears challenging, simpler fatty-acid membranes have been proposed to have formed the first cell-like structures^3,4^. However, fatty-acid membranes are unstable in the presence of divalent cations (e.g., Mg^2+^), which are, in turn, thought to be necessary for RNA folding, structure and function^5–7^. Considering that RNA is widely believed to have been a central component of the primordial genetic system^8,9^, such cell-like structures also need to be compatible with RNA catalysts (ribozymes); in particular, RNA polymerase ribozyme (RPR) to drive RNA replication^10,11^. However, although citrate has been shown to alleviate the destabilization of fatty-acid membranes by divalent cations^12^, it strongly inhibited RNA polymerization and ligation by ribozymes (RPR and L1 ligase), likely by chelating Mg^2+^ in an inaccessible form^13,14^.

Peptides are another type of biopolymer that could have existed and served as a biomolecular scaffold on the prebiotic earth^15–18^. We previously showed that simple cationic peptides (e.g., K_10_: KKKKKKKKKK) could reduce the strong Mg^2+^ dependency and enhance the function of RPR, likely by supporting interactions between negatively-charged RNA molecules to promote assembly of the RPR holoenzyme^13^. K_10_ was also found to stimulate the activity of the RNase P ribozyme, an ancient ribozyme conserved in all three domains of life^13,19^, indicating that similar cationic peptides might have functioned as general co-factors for numerous ribozymes. Various plausible pathways for the prebiotic synthesis of such charged peptides have been suggested^15,16^. For example, thermodynamic calculations predicted the prebiotic synthesis of positively/negatively-charged peptides on mineral surfaces^20,21^. Depsipeptides containing cationic proteinaceous amino acid residues (Lys, Arg) can be synthesized in dry-down reactions and mutually stabilize RNA^22,23^. Recently, the chemoselective synthesis of lysine-containing peptides was achieved even in aqueous solutions, by prebiotically-plausible chemistry^24^. All of these empirical observations, together with theoretical considerations (as well as analogies with extant biology), support the idea that simple peptides could have critically aided the emergence and function of RNA-based biosystems.

In this context, the ability of peptides to spontaneously form a wide variety of aggregates and liquid-liquid phase-separations (LLPSs) is of particular relevance, outlining another candidate pathway for the formation of prebiotic biomolecular assemblies^17,18,25–27^. Indeed, the hammerhead ribozyme (~30 nt) can perform its self-cleavage reaction in LLPSs containing positively-charged peptides (e.g., poly-L-lysine)^28,29^, and its activity is enhanced by the addition of a negatively-charged peptide (D_10_), presumably by loosening the tight RNA-polycation interactions^30^. Most strikingly, RPR can be assembled from shorter fragments by the ligation activity of an engineered hairpin ribozyme in coacervates formed with poly-L-lysine^31^. However, the activity of RPR itself was strongly inhibited by such cationic peptides when their concentrations were high enough to form RNA-peptide macromolecular aggregates^13^.

Alternatively, hydrophobic peptide assemblies could have formed easily even without RNA, as peptides can be synthesized under a wide variety of various prebiotic conditions^15,16^. Some of these peptides could have self-propagated and spontaneously assembled to form insoluble aggregates, creating a scaffold for the organization of other molecules^18^. For example, simple peptides with alternating hydrophobic and hydrophilic residues (e.g., Val-Lys repeats) can form amyloid structures^18,32^, and can also be synthesized in prebiotic chemistry by using peptides with complementary charges as templates^33^. Furthermore, such peptides and nucleic acids can mutually promote amyloid formation and hybridization^34^. However, the effects of such peptide aggregates and scaffolds on ribozyme functions have not been studied in detail.

Here, we report on the properties of an RNA-binding peptide (P43: AKKVWIIMGGS), which was selected de novo by phage display for binding to an RPR construct, Zc^35^. P43 forms insoluble aggregates and shows both stimulative and inhibitive effects on various ribozymes, depending on the Mg^2+^ concentration ([Mg^2+^]). We characterized these effects and found that P43 -unlike K_10_, which stimulates RPR only at low [Mg^2+^] and becomes inhibitory when RNA-peptide macromolecular aggregates are formed^13^ - can concentrate RNAs and stimulate the RPR activity on the peptide aggregates at high [Mg^2+^]. Furthermore, we showed that such regulations of the RPR activity can also be performed by a simpler hydrophobic-cationic peptide (K_2_V_6_: KKVVVVVV). We propose that such hydrophobic-cationic peptide aggregates could have captured dilute nucleic acids and accelerated RNA synthesis by RPR when the conditions became optimal during environmental fluctuations (e.g., influx of nutrition and metal ions) on the primordial earth.

## Results

### Selection of peptides to regulate the RPR activity

**Fig. 1:**
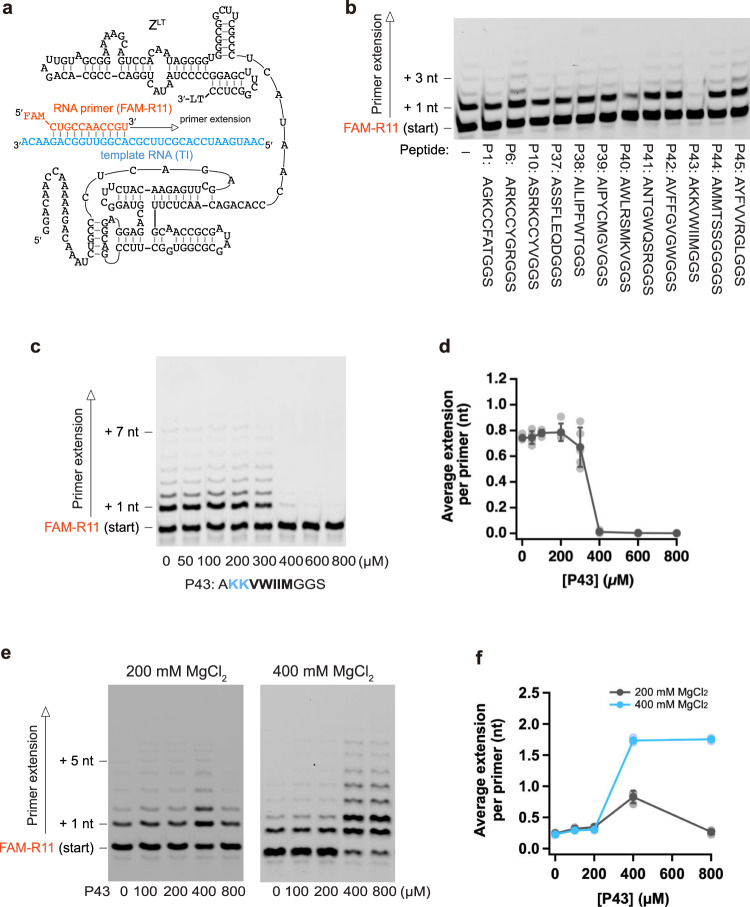
Selection of ribozyme-binding peptides. **a** Schematic depiction of an RNA polymerase ribozyme (RPR Z^LT^). 3′-LT is a 3′-extension sequence (GCGGCCGCAAAAAAAAAAGGCUUACC) used in the previous ribozyme selection for RPR Z^11^. **b**–**f** Primer extension by RPR Z^LT^ at low [Mg^2+^] was performed in 50 mM Tris•HCl ((**b**) pH 7.6, (**c**, **d**) pH 8.3) buffer containing 25 mM MgCl_2_, 8% PEG 6000, 0.4% DMSO and 500 µM of each NTP. The reactions were incubated at 17 °C for 7 days. Primer extension by RPR Z^LT^ at high [Mg^2+^] was performed in 50 mM Tris•HCl (pH 8.3) buffer containing 200 or 400 mM MgCl_2_, 0.4% DMSO and 500 µM of each NTP. The reactions were incubated at 17 °C for 3 days. **b** The peptide concentration was 0.4 mg/mL (P1: 400 µM; P6: 350 µM; P10: 350 µM; P38: 340 µM; P39: 380 µM; P40: 340 µM; P41: 360 µM; P43: 340 µM; P44: 420 µM; P45: 360 µM) except for P42, which was used as a 0.04 mg/mL (37 µM) mixture. **d**, **f** Data are presented as mean values ± S.D. (*N* = 5 (**d**) or 3 (**f**)).

Aiming to obtain new peptides that bind and regulate an RNA polymerase ribozyme (RPR Z^LT^, Fig. 1a), we first performed peptide selection by using the previously reported phage display protocol^36^. We prepared a phagemid library with seven randomized amino acid residues (AXXXXXXXGGS), and also synthesized a biotinylated RPR construct stabilized in the active conformation as the bait (RPR Zc, Supplementary Fig. 1a)^13,35^. We then performed four rounds of selection of the peptide library against the RPR Zc ribozyme immobilized on magnetic beads and sequenced the isolated phagemids to identify the selected peptide sequences after rounds 3 and 4 (Supplementary Fig. 1b). Roughly 70% of the selected sequences in rounds 3 and 4 contained a common motif (KCCF/Y). We stopped the selection experiment after round 4, since no additional motif was found in round 4. We then tested the effects of twelve selected peptides (three with the KCCF/Y motif and nine without it) on the activity of RPR under low [Mg^2+^] conditions (25 mM MgCl_2_) (Fig. 1b). As the primer extension by Z^LT^ is very slow at such low [Mg^2+^]^35^, 8% PEG6000 was added to the RPR reactions at 25 mM MgCl_2_ to accelerate the ribozyme by molecular crowding^13,37^. The tested peptides, with the exception of P42 and P43, were satisfactorily soluble under the tested conditions. The P42 peptide mostly precipitated as flocculent aggregates (Supplementary Fig. 2), and was added as a mixture containing precipitates at a lower concentration than the other peptides. In contrast, the hydrophobic-cationic peptide P43 (AKKVWIIMGGS) formed homogenous suspension of aggregates after overnight storage in the freezer (Supplementary Figs. 2, 3), and was tested as a suspension by mixing with the RPR reaction solution. While most of the peptides did not show any significant effects on the RPR activity, P43 strongly inhibited the activity of RPR at 25 mM MgCl_2_. When we varied the peptide concentration, P43 did not affect the ribozyme activity at low concentrations (50–200 µM), while relatively large amounts (≥400 µM) almost completely inhibited RPR (Fig. 1c, d).

Interestingly, when we tested the P43 peptide at higher [Mg^2+^], it had totally different effects on the RPR activity (Fig. 1e, f). At 200 mM MgCl_2_, 400 µM P43 increased the RPR activity roughly three-fold, while a higher concentration of the peptide (800 µM) almost canceled the effect. At 400 mM MgCl_2_, the RPR activity was increased more robustly (~8-fold) in the presence of 400–800 µM P43. We also observed the stimulation of the RPR activity by P43 under conditions with 25 mM MgCl_2_ and 800 mM KCl (Supplementary Fig. 4) and in the concentrated eutectic ice phases in frozen samples (Supplementary Fig. 5), although the effects were smaller than in the reaction at 400 mM MgCl_2_. These observations indicate that P43 can enhance RPR at high ionic concentrations, quite unlike the simple cationic K_10_ peptide, which can stimulate RPR only at low [Mg^2+^], where the activity of RPR Z^LT^ was severely limited^13^. It was, therefore, unexpected and compelling that P43 could further enhance the RPR activity under the optimal conditions for the ribozyme (200–400 mM MgCl_2_). Thus, in this study, we sought to analyze the properties of P43 and its analogues in more detail.

### P43 forms insoluble aggregates and captures RNA in a size-dependent manner

**Fig. 2:**
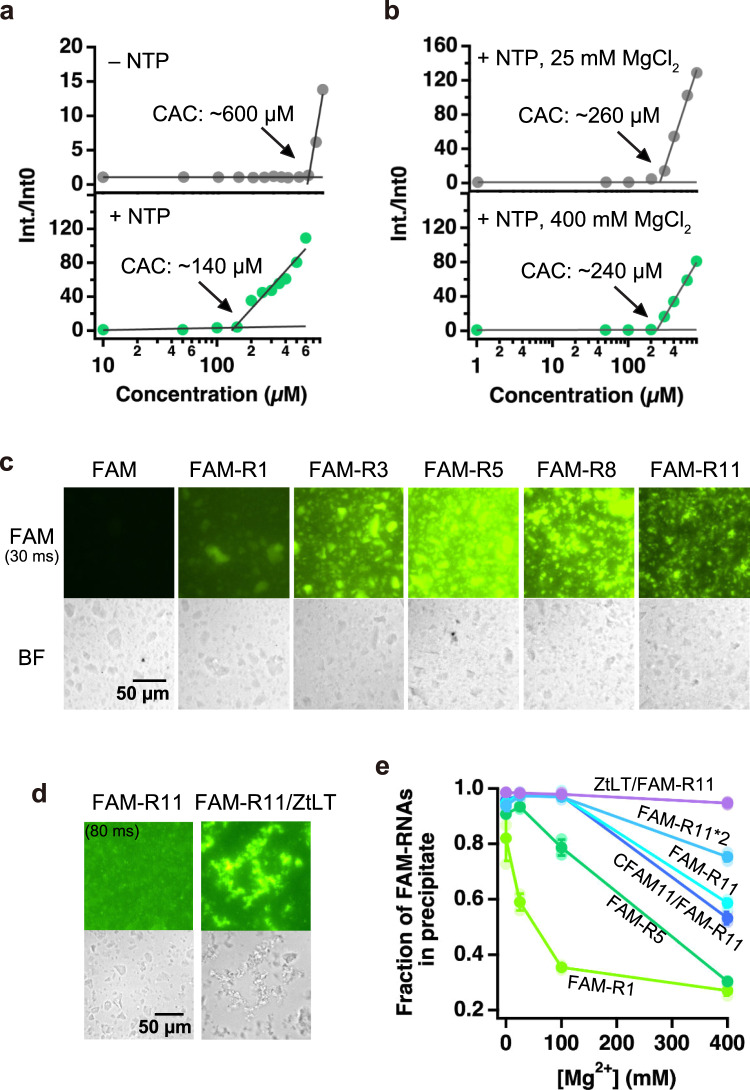
Aggregates of the P43 peptides. **a** The CAC of P43 in 0.4% DMSO in the absence or presence of 500 µM NTPs. **b** The CAC of P43 in 50 mM Tris•HCl (pH 8.3) buffer containing 500 µM NTPs, 0.4% DMSO, and 25 mM or 400 mM MgCl_2_. **c** Microscopic analysis of the P43 aggregates (4 mM, 2% DMSO) mixed with fluorescently-labeled RNAs (20 µM FAM-R1–R11) in 0 mM MgCl_2_. The samples were observed with bright-field and fluorescence-filter setups (FAM, exposure time = 30 ms). **d** Microscopic analysis of the P43 aggregates (4 mM, 2% DMSO) mixed with fluorescently-labeled RNAs (0.5 µM FAM-R11 and Zt^LT^/FAM-R11) at 0 mM MgCl_2_. The samples were observed with bright-field and fluorescence-filter setups (FAM, exposure time = 80 ms). **e** The fraction of fluorescently-labeled RNAs (0.25 µM) in the precipitations with 800 µM P43 in 50 mM Tris•HCl (pH 8.3) buffers, containing 0.4% DMSO and 0, 25, 100 or 400 mM MgCl_2_. The samples were separated by centrifugation (13000 rpm, 3 min). Error bars represent S.D. (*N* = 3).

As P43 formed visible aggregates in the stock sample (Supplementary Fig. 3), we measured its critical aggregation concentration (CAC) and evaluated the effects of NTPs, by using 8-anilino-1-naphthalenesulfonic acid (ANS) as a fluorescent probe to detect aggregate formation (Fig. 2a, Supplementary Fig. 6). In the absence of NTPs, P43 formed aggregates at ~600 µM, while in the presence of NTPs, it formed aggregates at ~140 µM. The negatively-charged triphosphate moiety of NTPs might have reduced the repulsion between the peptide molecules containing two positively-charged lysine residues and enabled aggregation at lower peptide concentrations. In the reaction buffer for RPR containing NTPs and 25/400 mM MgCl_2_, the CACs of P43 were ~260/240 µM, respectively (Fig. 2b, Supplementary Fig. 7). This is consistent with the observation that concentrations of P43 at 200 µM or less did not affect the RPR activity significantly. Thus, the activity modulation of RPR was correlated to the P43 aggregate formation, suggesting that the peptide aggregates act as an RNA-binding scaffold.

To investigate the structure of the P43 aggregates, we stained them with Congo Red dye (Supplementary Fig. 8), which revealed apple-green birefringence, an indication of amyloid structures. This observation is consistent with the presence of β structures in the P43 aggregates suggested by X-ray diffraction, CD spectra, and electron microscopy observations (Supplementary Figs. 9–11). One might speculate that the formation of such regular structures by P43 underlies at least some of its effects on RPR. Unlike the flexible K_10_ peptide that inhibits the RPR activity at high peptide concentrations^13^ a regular scaffold might be helpful to suppress inhibitive, irregular interactions between RNA and peptides (Fig. 1e, f). Furthermore, the fact that the hydrophobic-cationic P43 peptide could still enhance the RPR activity at high [Mg^2+^], where the purely cationic K_10_ peptide lost its effect^13^, might be due to the multivalency of the cationic charges within the P43 aggregates that enables interactions with RNA even at high ionic strength.

Next, we sought to determine whether RNA binds to the P43 aggregates. To this end, we performed a microscopic analysis of the P43 aggregates in the presence and absence of fluorescently-labeled RNA molecules (FAM-R1–R11, Supplementary Table 2). A higher peptide concentration (4 mM P43) than that in the RPR reaction was applied to observe the aggregates clearly by optical microscopy in the absence of NTPs and MgCl_2_ (Fig. 2c). The P43 aggregates appeared the same regardless of the presence of RNA when viewed under bright field, while the fluorescence microscopy observation clearly showed that the RNA molecules bound to the P43 aggregates. When we compared the fluorescent signals from 1–5 nt RNAs (20 µM FAM-R1, FAM-R3, FAM-R5), the signal increased with the RNA size, indicating that the P43 peptide can interact more stably with longer RNA molecules. However, with 5–11 nt RNAs (20 µM FAM-R5, FAM-R8, FAM-R11), the fluorescent signal rather decreased as the RNA size increased. This apparent contradiction did not occur at lower RNA concentrations (0–5 µM FAM-R1, FAM-R5, FAM-R11, Supplementary Fig. 12). As the RNA concentration increased (10–40 µM), only the fluorescent signal of FAM-R11 became saturated gradually, suggesting that the aggregate surface was saturated at lower RNA concentrations by the longer RNA molecules. We also applied an RPR construct conjugated to the RNA template and stably hybridized with FAM-R11 (Zt^LT^/FAM-11, Supplementary Table 2)^35^ at a lower RNA concentration (0.5 µM, Fig. 2d). The fluorescent signal of Zt^LT^/FAM-11 was more robust than that of FAM-R11 alone. Interestingly, the P43 aggregates became larger in the presence of FAM-11/Zt^LT^. Z^LT^ (0.25 µM) and another ribozyme (HH35) could also reduce the P43 concentration required to form aggregates, even in the absence of NTPs (Supplementary Fig. 13). Thus, longer RNAs can bind to the P43 aggregates more robustly and further seed the growth of the aggregates.

Since P43 shows different effects on the RPR activity at 25 mM and 400 mM MgCl_2_, we also investigated the effect of the MgCl_2_ concentration on the interactions between P43 and RNA. We measured the RNA fractions in precipitations with P43, using samples at the same concentrations as in the RPR reaction experiments (800 µM P43 and 0.25 µM RNA, Fig. 2e, Supplementary Fig. 14). In addition to FAM-R1, FAM-R5, FAM-R11, and Zt^LT^/FAM-R11, a 22 nt ssRNA (FAM-R11*2) and an 11 bp dsRNA (CFAM11/FAM-R11) were tested (Supplementary Table 2). As the MgCl_2_ concentration increased (100–400 mM), less RNA bound to the P43 aggregates, indicating that high ionic strengths decreased the binding affinity between the peptide and RNA. When we compared ssRNA molecules (FAM-R1, FAM-R5, FAM-R11, and FAM-R11*2), longer RNAs could bind more stably to P43 at high [Mg^2+^]. The same lengths of ssRNA and dsRNA (FAM-R11 and CFAM11/FAM-R11) showed similar affinities to the peptide aggregates. Zt^LT^/FAM-R11, the largest RNA construct tested, is mostly bound to the aggregates even at 400 mM MgCl_2_. Thus, although high [Mg^2+^] can significantly decrease the affinity between P43 and short RNAs (like FAM-R1–R11), long RNAs (like RPR) can stably remain on the aggregate surfaces at various [Mg^2+^].

### The effects of P43 depend on both cationic and hydrophobic amino acid residues

**Fig. 3:**
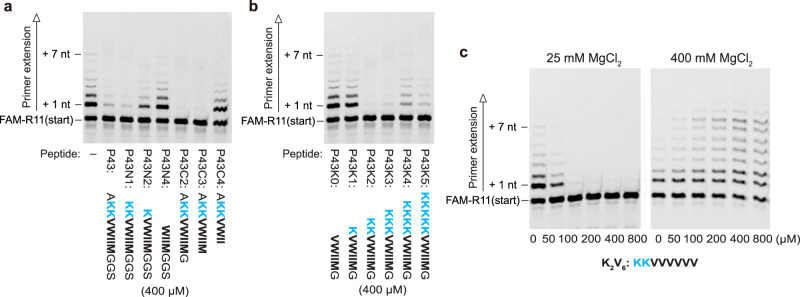
Inhibition of the RPR activity by P43 variants. Primer extension by RPR Z^LT^ at 25 mM MgCl_2_ was performed in 50 mM Tris•HCl (pH 8.3) buffer containing 25 mM MgCl_2_, 8% PEG 6000, 0.4% (**a**, **b**) or 0.8% (**c**) DMSO and 500 µM of each NTP. The reactions were incubated at 17 °C for 7 days. Primer extension by RPR Z^LT^ at 400 mM MgCl_2_ was performed in 50 mM Tris•HCl (pH 8.3) buffer containing 400 mM MgCl_2_, 0.8% DMSO and 500 µM of each NTP. The reaction was incubated at 17 °C for 3 days.

To identify the parts of the P43 peptide that were essential for its properties, we first tested the inhibitory effects of truncated variants of P43 at 25 mM MgCl_2_ (400 µM peptides, Fig. 3a). Although the N-terminal and C-terminal amino acid residues that were fixed in the phage display library (Ala1, Gly9, Gly10, and Ser11) were dispensable for the inhibitory effect (P43N1, P43C2, and P43C3), further truncation decreased the inhibitory effect to a large extent (P43N2) or completely (P43N4, and P43C4). We also confirmed that all effective variants formed aggregates with NTPs (Supplementary Fig. 15a, b). These results indicated that both the cationic part (Lys2–Lys3) and hydrophobic part (Val4–Met8) of P43 are essential for this effect.

As the interaction between RNA and P43 seemed to be mostly electrostatic and mediated by the cationic part (Lys2–Lys3), we next tested P43 variants with different numbers of lysine residues at 25 mM MgCl_2_ (400 µM peptides, Fig. 3b). The P43 variants with zero or only one lysine residue showed almost no inhibitory effect on RPR (Fig. 3b, P43K0, and P43K1). Although we expected that the P43 variants with more than two lysine residues (P43K3, P43K4, P43K5) would interact and inhibit RPR more strongly than the variant with two lysine residues (P43K2), the inhibitory effect rather decreased when the number of lysine residues was increased from two (Fig. 3b). At a lower concentration (200 µM), P43K3–K5 did not show significant inhibitive effects, while P43K2 still inhibited the activity of RPR (Supplementary Fig. 16). P43K3 was also less prone to aggregate than P43K2 (Supplementary Fig. 15c). At 400 µM, P43K4 and P43K5 did not form aggregates even in the presence of NTPs, while they still aggregated in the mixture with a longer RNA (0.25 µM Z^LT^) (Supplementary Fig. 17). Thus, while electrostatic interactions likely mediate the binding of RNA to the peptides, the propensities of the peptides to aggregate are important factors for the inhibitory effect.

It is also important to note that 200 µM P43K2 could inhibit RPR (Supplementary Fig. 16), while the original P43 peptide required higher concentrations to have the same effect (Fig. 1c, d), although their sequence difference is only the absence/presence of dispensable residues (Ala1, Gly10, and Ser11). We determined that the CAC of P43K2 at 25 mM MgCl_2_ is about 100 µM (Supplementary Fig. 18), which is significantly smaller than the CAC of P43. These observations also indicate that P43 and its variants modulate the activity of RPR in the aggregated forms.

Next, we tested another hydrophobic-cationic peptide with a simplified sequence (K_2_V_6_: KKVVVVVV) to investigate whether the inhibitory and stimulative effects on the RPR activity are a general feature of such peptides (Fig. 3c). In the presence of 100 µM or more K_2_V_6_, the RPR activity was strongly inhibited at 25 mM MgCl_2_, and strongly enhanced at 400 mM MgCl_2_. The CAC of K_2_V_6_ with NTPs was ~80 µM (Supplementary Fig. 19). Thus, the inhibitory and stimulative effects do not appear to depend on a specific sequence and would reside in a wide variety of hydrophobic-cationic peptides that form aggregates capable of binding RNA.

Other hydrophobic-cationic peptides with similar sequences (K_3_V_6_: KKKVVVVVV; K_5_V_6_: KKKKKVVVVVV; K_5_V_9_: KKKKKVVVVVV; KGKV_6_: KGKVVVVVV) showed comparable inhibitory effects at 25 mM MgCl_2_, although the required peptide concentrations and strengths of the effects varied between the designs (Supplementary Fig. 20, top). At 400 mM MgCl_2_, K_3_V_6_ and KGKV_6_ showed significant stimulatory effects, while the effect of K_5_V_6_ was weaker than the others (Supplementary Fig. 20, bottom). K_5_V_6_ was also less aggregation prone. Even in the presence of 0.25 µM Z^LT^, it aggregated only at 25 mM MgCl_2_ and did not form any aggregates at 400 mM MgCl_2_ (Supplementary Fig. 21), indicating that the excessive lysine residues increase the solubility of the peptide and hinder its aggregation. In contrast, a peptide with the same number of lysine residues but a longer hydrophobic tail (K_5_V_9_) aggregated at both 25 mM and 400 mM MgCl_2_ (Supplementary Fig. 21), and recovered the strong stimulatory effect on the RPR activity at 400 mM MgCl_2_ (50–200 µM K_5_V_9_, Supplementary Fig. 20). However, 800 µM K_5_V_9_ rather inhibited the RPR activity even at 400 mM MgCl_2_. With its larger number of lysine residues, the K_5_V_9_ peptide might interact with RNA more strongly than K_2_V_6_, K_3_V_6,_ and KGKV_6_, and thereby inhibited the ribozyme as in the lower MgCl_2_ conditions. The hydrophobic part of K_5_V_9_ is essential for such strong interactions with RNA, as a simple cationic peptide without hydrophobic residues (K_5_: KKKKK) did not show any significant effects at 25 and 400 mM MgCl_2_ (Supplementary Fig. 22). These results indicate that the balance between the numbers of hydrophobic and cationic residues is the key for the P43-like properties.

### The mechanism of the RPR activity modulation by P43

**Fig. 4:**
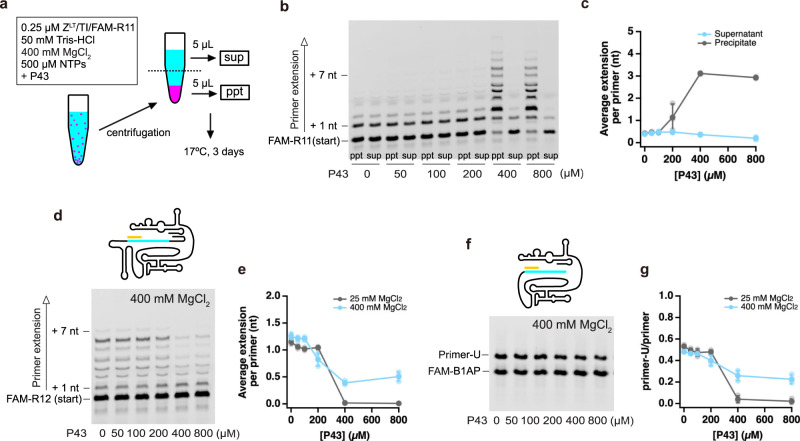
Modulation of the RPR activity by the P43 aggregate in various experimental conditions. **a** Schematic depiction of the experimental procedure. **b**, **c** Primer extension by RPR Z^LT^ was performed in 50 mM Tris•HCl (pH 8.3) buffer containing 400 mM MgCl_2_, 0.4% DMSO and 500 µM of each NTP. The reactions were incubated at 17 °C for 3 days. **d**–**g** P43 effect on the activities of RPR mutants, Zt^LT^ (**d**, **e**) and Zc (**f**, **g**). Primer extension was performed in 50 mM Tris•HCl (pH 8.3) buffer containing 25 or 400 mM MgCl_2_, 0.4% DMSO and 500 µM of each NTP (**d**, **e**) or 2 mM UTP (**f**, **g**). The reactions at 25/400 mM MgCl_2_ were incubated at 17 °C for 60/30 min (**d**, **e**) or 10/5 min (**f**, **g**). **c**, **e**, **g** Data are presented as mean values ± S.D. (*N* = 3).

As the inhibitory/stimulative effects on the activity of Z^LT^ appeared to be correlated to the peptide aggregate formation, we next tried to differentiate the RPR activities in the aggregates and the supernatant. After preparing reaction mixtures containing 400 mM MgCl_2_, we divided them into the top half (supernatant) and bottom half (containing aggregates) fractions by centrifugation (13000 rpm, 3 min), and then incubated each fraction separately (Fig. 4a–c). At the P43 concentrations lower than its CAC (0–200 µM P43), there was no detectable difference between the top and bottom fractions. At 400–800 µM P43, the RPR activity in the bottom fraction was even higher than the case without the separation procedure (Figs. 1f and 4c). In contrast, the RPR activity in the top fraction was slightly decreased. When we divided the samples into supernatant and precipitate after the RPR reaction, no difference between the average primer extensions in the two fractions was observed (Supplementary Fig. 23a–c), indicating that the RNA primer (and template) existed in dynamic equilibrium between the free and bound states at 400 mM MgCl_2_. At 25 mM MgCl_2_, most of the RNA primers were precipitated at 400–800 µM P43, with no detectable extension (Supplementary Fig. 23d). These results clearly indicate that the modulation of the RPR activity occurs on the surfaces of the P43 aggregates.

To understand how aggregation affects the activity of RPR in more detail, we performed a time-course analysis of the Z^LT^ reaction and monitored the integrity of RPR after reactions in the presence of 0, 100, and 400 µM P43 (Supplementary Fig. 24). Inhibition/enhancement of the RPR activity was already observed on day 1. The ribozyme degraded at almost the same speed in the absence/presence of P43. Thus, the P43 peptide does not have any detectable effect on the chemical stability of the ribozyme.

As the docking process between the ribozyme and the RNA primer/template is the rate-limiting step for Z^LT^^35^, we also tested the effect of P43 on the activities of Zt^LT^ and Zc, which are conjugated with the RNA template by long and short linkers, respectively (Fig. 4d–g, Supplementary Fig. 25)^13,35^. Zt^LT^ and Zc were inhibited by the P43 peptide at 25 mM Mg^2+^, in the same way as Z^LT^ (Fig. 4e, g, Supplementary Fig. 25b, c). However, they were still inhibited slightly even at 400 mM Mg^2+^ (Fig. 4d–g), where Z^LT^ was strongly enhanced (Fig. 1e, f). Thus, the stimulation of the Z^LT^ activity by P43 in high Mg^2+^ conditions was probably induced by the accretion of RNA on the aggregate surfaces, leading to the accelerated assembly of the RPR holoenzyme. In contrast, the inhibition of the RPR activity by P43 might be caused by the tight interactions between RNA and the peptide at low [Mg^2+^], resulting in the distortion of the active conformation of RPR. Higher [Mg^2+^] might release the RNA from such strong interactions with the P43 aggregates (Fig. 2e), and thus the inhibitory effect can also be weakened.

### Reactivation of captured RPR on the P43 aggregates

**Fig. 5:**
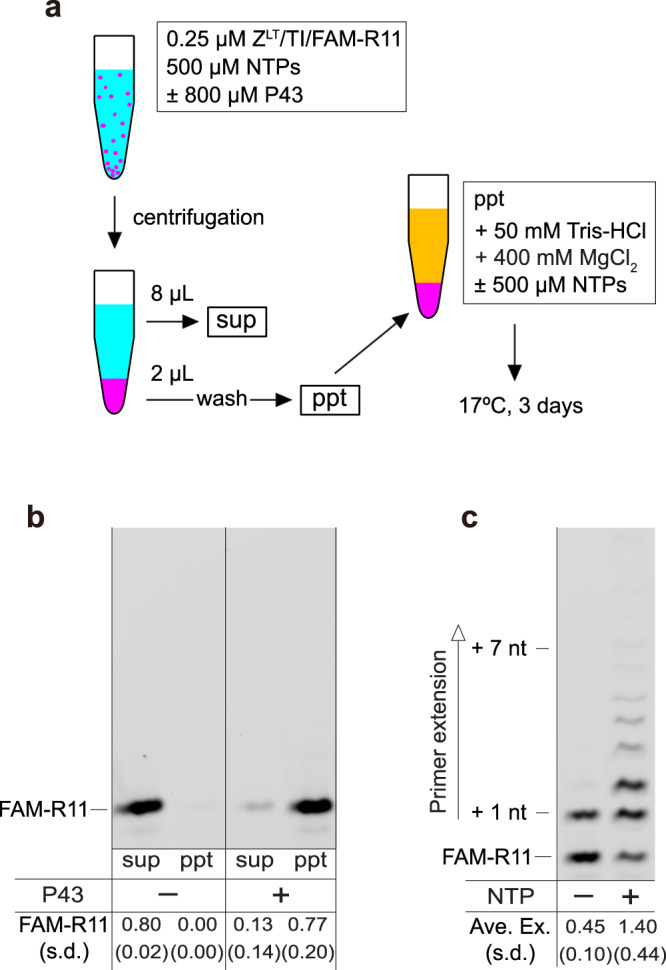
Reactivation of the captured RPR in high MgCl_2_ conditions. **a** Schematic depiction of the experimental procedure. **b** The fluorescently-labeled primer contained in the supernatant and precipitate samples. The amount of FAM-R11 in the supernatant without P43 was normalized as 0.80, by assuming that 80% of the RNA from the 10 µL sample was recovered in the 8 µL supernatant. **c** Co-precipitation of RPR and P43, incubated in 50 mM Tris•HCl (pH 8.3) buffer containing 400 mM MgCl_2_, ± 500 µM of each NTP at 17 °C for 3 days. **b**, **c** Mean values and S.D. are presented below the gel images (*N* = 3).

As [Mg^2+^] can affect the interactions between the P43 aggregates and RNA, we tested whether RPR captured on the P43 aggregates in low ionic strength conditions could be reactivated in a high ionic strength environment (Fig. 5a). First, we prepared RNA-peptide premixtures containing Z^LT^, the RNA template/primer, and NTPs in the presence and absence of 800 µM P43. Next, we centrifuged the mixtures to divide them into supernatants and precipitates (13000 rpm, 1 min). To remove unbound materials, we gently washed the precipitates with H_2_O three times without disrupting the aggregates collected at the bottom of the reaction tubes. The fluorescently-labeled RNA primer was found with the aggregates in the presence of the P43 peptide (Fig. 5b, right), while it was left in the supernatant in the absence of P43 (Fig. 5b, left). Finally, we dispersed the P43-RNA precipitates into a reaction buffer containing 400 mM MgCl_2_ with or without additional 500 µM NTPs to form a suspension again, and incubated them for 3 days at 17 °C. In the case of the reaction buffer with additional NTPs, the average primer extension by RPR was 1.40 ± 0.44 nt (Fig. 5c, right), which was comparable to the RPR activity under the same conditions without the centrifugation/resuspension processes (the average primer extension at 400 mM MgCl_2_ and 800 µM P43 was 1.75 ± 0.02 nt, Fig. 1e, f). These results indicate that most of the RPR was captured on the P43 aggregates and could be reactivated in the high Mg^2+^ buffer. Even when we resuspended the co-precipitates of P43 and RPR in reaction buffer without additional NTPs, RPR showed detectable activity (Fig. 5c, left), although it was significantly weaker than the case with additional NTPs. The absorbances at 260 nm and 280 nm (A_260_ and A_280_) of the supernatant from the mixture of 800 µM P43 and 500 µM of each NTP indicated only ~10% of NTPs (50 µM each) co-precipitated with the peptide (Supplementary Fig. 10c). In contrast, most of the RNA molecules were captured on the P43 aggregates (Fig. 5b). Thus, hydrophobic-cationic peptide aggregates can accumulate RNA (and NTPs to a lesser extent) on their surfaces in low ionic strength conditions and reactivate/enhance ribozyme activities in high ionic strength conditions. Such a mechanism might also have supported the accumulation and activation of ribozymes in the fluctuating environments of the prebiotic earth (e.g., temperature, solute concentrations)^38–41^.

### Different ribozymes are affected by P43 in different ways

**Fig. 6:**
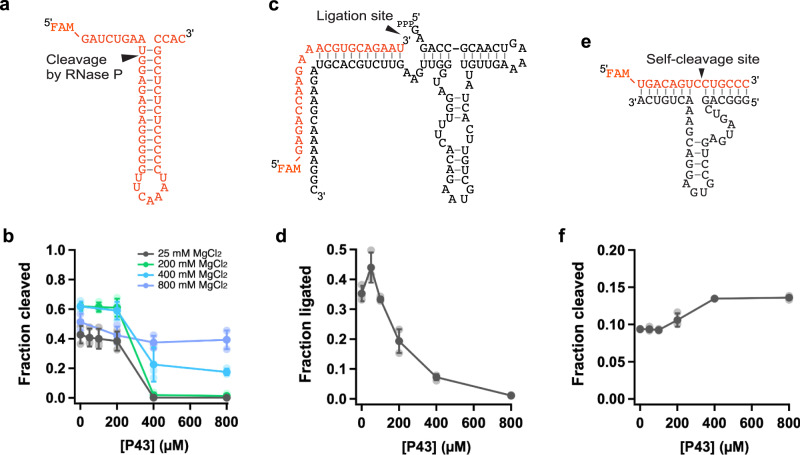
Activities of various ribozymes in the presence of P43. **a** Schematic depiction of the substrate for the RNase P ribozyme (pATSerUG). **b** The substrate cleavage reaction by RNase P in the presence of P43 and different MgCl_2_ concentrations. The reactions were incubated in 50 mM Tris•HCl (pH 8.0) buffer containing 25 mM MgCl_2_ at 37 °C for 10 min, or in 50 mM Tris•HCl (pH 8.0) buffer containing 200–800 mM MgCl_2_ and 0.4% DMSO at 37 °C for 2.5 min. **c** Schematic depiction of F1* ligase. **d** The self-ligation reaction by F1* ligase was performed in 50 mM Tris•HCl (pH 8.3), 25 mM MgCl_2_, 0.4% DMSO at 25 °C for 20 s. **e** Schematic depiction of the trans-cleaving hammerhead ribozyme. **f** The substrate cleavage reaction by the hammerhead ribozyme in 50 mM Tris•HCl (pH 8.3), 25 mM MgCl_2_, 0.4% DMSO. The reactions were incubated at 25 °C for 20 s. **b**, **d**, **f** Data are presented as mean values ± S.D. (*N* = 3).

As the P43 peptide was shown to interact with RNA molecules non-specifically (Fig. 2), we also investigated its effects on the activities of a few different ribozymes (Fig. 6 and Supplementary Figs. 26–29). First, we tested the activity of the ribozyme component of RNase P (377 nt), which cleaves the 5′-extension of pre-tRNA to produce matured tRNA^42^. In this experiment, we prepared an RNA helix mimicking the 5′-extension and the acceptor stem of pre-tRNA (pATSerUG) as the substrate for RNase P (Fig. 6a)^43–45^. In low MgCl_2_ conditions (25 mM), the activity of RNase P to cleave the 5′-extension of pATSerUG was inhibited in the presence of 400–800 µM P43, similarly to RPR (Fig. 6b, Supplementary Fig. 26a). We next investigated the RNase P activity under higher MgCl_2_ conditions (Fig. 6b, Supplementary Fig. 26b–d). P43 strongly inhibited the RNase P activity even at 200 mM MgCl_2_. At 400 mM MgCl_2_, P43 still inhibited RNase P ribozyme but in a more moderate way, leaving significant activity in the presence of 400–800 µM P43. At 800 mM MgCl_2_, P43 finally lost the significant inhibitory effect, indicating that the tight interaction between RNase P and P43 was mitigated in the high [Mg^2+^] conditions. The effect of P43 on RNase P was not significantly influenced by the presence of NTPs (Supplementary Fig. 27). RNase P was not enhanced by P43 even at high [Mg^2+^], unlike Z^LT^, presumably because the complex between RNase P and its substrate is quite stable^44^.

We also tested the effect of P43 on the activity of two shorter ribozymes, F1* ligase ribozyme (Fig. 6c, 81 nt ribozyme strand + 22 nt substrate strand)^46^ and the trans-cleaving hammerhead ribozyme (Fig. 6e, 35 nt ribozyme strand + 14 nt substrate strand)^47^ in the presence of the P43 peptide. We found that even in a low MgCl_2_ condition (25 mM), the self-ligation activity of F1* ligase was not completely inhibited by 400 µM P43 (Fig. 6d, Supplementary Fig. 28). The activity of the hammerhead ribozyme was slightly enhanced in the presence of 400–800 µM P43 when we first annealed the ribozyme and substrate, and then mixed this complex with the reaction buffer containing the peptide at 25 mM MgCl_2_ (Fig. 6f, Supplementary Fig. 29a). However, such enhancement was not observed when the hammerhead ribozyme and substrate were mixed in the presence of P43 (Supplementary Fig. 29b, c). Thus, P43 modulates the activities of various ribozymes in different ways (depending on experimental procedures), although the detailed mechanisms remain to be investigated.

## Discussion

We have reported on a new RNA binding peptide, P43 (AKKVWIIMGGS), selected by phage display. The P43 peptide revealed some general principles of the interaction between RNA and cationic-hydrophobic peptides and the resulting modulation of ribozyme activities. P43 forms aggregates containing amyloid structures and influences the activities of various ribozymes. The interactions between the RNA and the P43 peptide became weaker with increasing the concentration of MgCl_2_ (Fig. 2e). In low ionic strength conditions (25 mM MgCl_2_), the P43 peptide strongly inhibited RNA primer extension by RPR (Fig. 1c, d). At high concentrations of MgCl_2_ (≥ 200 mM), the peptide aggregates rather enhanced the activity of RPR (Fig. 1e, f), probably by promoting the assembly of the RPR holoenzyme on the aggregate surfaces without strong distortion or unfolding of the ribozyme structure. Furthermore, the RPR captured on the peptide aggregates in low ionic strength conditions (0 mM MgCl_2_) could be reactivated by resuspension in high ionic strength conditions (400 mM MgCl_2_) (Fig. 5). Considering that a simplified K_2_V_6_ peptide showed very similar inhibitory and stimulatory effects, like P43 (Fig. 3c), a wide variety of hydrophobic-cationic peptides likely have the potential to similarly capture and inhibit/enhance ribozyme functions.

Our results also suggest the potential beneficial roles of hydrophobic-cationic peptide aggregates in the emergence of RNA-based life. On the primordial earth, the environment surrounding the primitive biopolymers would have inevitably fluctuated (e.g., temperature, solute concentrations, ionic strength), and the capacity of biopolymers to survive under a variety of such conditions would have likely been critical^38–41,48–55^. Hydrophobic-cationic peptides could have become concentrated as aggregates without dissipating and such aggregates could have accumulated scarce nucleotides and RNA molecules on their surfaces and prevented them from being washed away (e.g., by rainfall, flowing water). The same process would also have served as a selection for longer RNA molecules, as they bound to the P43 peptide aggregates more stably than shorter ones (Fig. 2c–e). When the environment became more optimal for the RPR activity (high [Mg^2+^]); for example, by partial evaporation, influxes of different streams or seasonal/diurnal freezing, the peptide aggregates could then have enhanced RNA synthesis by RPR. Such environments could have also supported non-enzymatic biopolymer formation^48–55^. Thus, hydrophobic-cationic peptide aggregates could have enriched longer and functional RNA molecules in the fluctuating environments, unlike the LLPS structures formed with soluble cationic peptides and RNA that can be easily disassembled under high ionic strength conditions^56^. Therefore, RNA-binding peptide aggregates could have promoted the emergence of RNA-based life by preventing dispersion of RNA in harsh prebiotic environments, before the first cell-like structure was established.

Furthermore, such processes might have operated concurrently with the evolution from simple peptides to functional proteins. Peptides containing amyloid structures have been suggested to have given rise to ancient proteins with more complicated 3D structures^18^. Prebiotic peptide ligation in water, recently reported by Canavelli et al.^24^, might have supported the transition from short peptides into functional proteins by allowing protein folding to occur in the same aqueous environment. We have recently reconstructed the ancient β-barrel fold at the core of proteinaceous RNA polymerase by the homodimerization of a peptide with only seven amino acid types^57^. Valine and lysine are the two richest amino acid types and together constitute more than half of the peptide (eight lysine and fourteen valine residues in the 43 a.a. peptide), suggesting that its ancestors (like K_2_V_6_) might have both captured RNA and enhanced RNA polymerization by ribozymes.

## Methods

### Preparation of RNAs and peptides

The DNA and RNA sequences used in this study are listed in Supplementary Tables 1 and 2. Z^LT^, Zc, and RNase P ribozymes were synthesized by in vitro transcription, as previously described^11,13,35^. The 3′-end of Zc was then biotinylated by using the periodate oxidation method^58^. Briefly, Zc was incubated in 66 mM sodium acetate (pH 4.5) with 5 mM NaIO_4_ on ice for 45 min in the dark. After the incubation, the ribozyme was purified by isopropanol precipitation. Zc was then incubated in 20 mM sodium acetate (pH 6.1), 0.2% SDS, 70% DMSO, and 7 mM biotin-LC-hydrazide (Thermo Fisher Scientific) overnight at 23 °C in the dark. Finally, the labeled Zc ribozyme was purified by ethanol precipitation.

The sequence encoding F1* ligase was amplified by PCR using gF1*_C, gF1*_F, and gF1*_R as the template and primers. The 5′-triphosphorylated L1* ligase was then transcribed with an in vitro transcription kit (MEGAshortscript T7 transcription kit, Invitrogen). Chemical synthesis and HPLC purification of the hammerhead ribozyme and fluorescently-labeled RNA substrates (FAM-R1–11, pATSerUG, substrate strands for F1* ligase and the hammerhead ribozyme) were performed by Japan Bio Services. pATSerUG was further gel-purified to remove contaminants.

All peptides tested in this report were supplied by Japan Bio Services. We dissolved the peptides in 100% DMSO and then diluted them with H_2_O to prepare stock solutions or suspensions.

### Peptide selection

To select peptides that bind to RPR, we applied the previously-reported phage display method^36^, omitting the chemical cyclization procedure of the peptide libraries. Briefly, we first prepared the peptide library with seven randomized amino acid residues (AXXXXXXXGGS) fused to pIII of the pHEN1 phagemid vector. The phagemid library was then expressed and purified by PEG precipitation. We performed four rounds of biopanning of the peptide library against the biotinylated Zc ribozyme immobilized on magnetic beads (Dynabeads M-280 Streptavidin for rounds 1, 3, 4; Dynabeads M-280 coated with NeutrAvidin for round 2). The magnetic beads were mixed with the biotinylated ribozyme, blocked with ROTI-Block, and washed with wash buffer, containing 20 mM Tris-HCl (pH 7.4), 100 mM NaCl, 20 mM MgCl_2_, and 0.1% Tween 20, using a KingFisher mL purification system. The beads were then mixed with the phagemid library, incubated at 4 °C for 30 min, and washed with the washing buffer. Subsequently, the magnetic beads were added to TG1 cells (Lucigen) for infection and plating. Finally, we sequenced ~30 and ~50 phagemids after the third and fourth rounds of biopanning, respectively. The sequences without any stop codon are listed in Supplementary Fig. 1b.

### Activity test of RNA polymerase ribozyme

Initially, RPR Z^LT^ was annealed with the fluorescently-labeled RNA primer (FAM-R11) and RNA template (TI) in H_2_O (50 °C for 5 min, 17 °C for 10 min). The ribozyme complex was then mixed with the extension buffer, so that each reaction contained 0.25 µM Z^LT^/FAM-R11/TI, 50 mM Tris•HCl (pH 7.6 or 8.3), 25–400 mM MgCl_2_, 0 or 8% PEG 6000, 0.4% DMSO, 500 µM of each NTP, and the peptides. The samples were incubated at 17 °C for 3 or 7 days. PEG 6000 was mixed in the low [Mg^2+^] reactions as a molecular crowding agent to increase the reaction rate. In the cases of RPR Zt^LT^ and Zc, the ribozymes were annealed with the fluorescently labeled RNA primers (FAM-R12 for Zt^LT^ and FAM-B1AP for Zc). The primer extension was then performed in 50 mM Tris•HCl (pH 8.3) buffer containing 25 or 400 mM MgCl_2_, 0.4% DMSO and 500 µM of each NTP (Zt^LT^) or 2 mM UTP (Zc). The reactions were incubated at 17 °C for 60/30 min (Zt^LT^) or 10/5 min (Zc) at low / high [Mg^2+^] respectively. The reactions were stopped by adding 3–6 volumes of stop buffer (8 M urea, 80 mM EDTA, and 10 µM TC2). After heat treatment (94 °C, 5 min), the samples were resolved by urea-PAGE gels (20% polyacrylamide, 8 M urea). The gels were analyzed using an Amersham Typhoon scanner (GE Healthcare, Amersham Typhoon 1.1.0.7).

### Activity test of RNase P ribozyme

First, *E. coli* RNase P ribozyme and pATSerUG were annealed in 1 mM EDTA (pH 8.0), by heating the samples at 70 °C for 5 min and gradually decreasing the temperature to 12 °C. The ribozyme complex was then mixed with the reaction, so that each reaction contained 0.25 µM RNase P/pATSerUG, 50 mM Tris•HCl (pH 8.0), 25–800 mM MgCl_2_, 0.4% DMSO, and the peptides. The samples were incubated at 37 °C for 2.5–10 min. The RNA cleavage reactions by RNase P were stopped by adding 3–12 volumes of stop buffer (8 M urea, 80 mM EDTA). After heat treatment (94 °C, 5 min), the samples were resolved on urea-PAGE gels (10–15% polyacrylamide, 8 M urea). The gels were analyzed using an Amersham Typhoon scanner.

### Activity test of F1* ligase

F1* ligase was annealed in H_2_O with the labeled substrate strand, F1*sub, by heating the samples at 70 °C for 5 min and gradually decreasing the temperature to 12 °C. Next, the ribozyme complex was mixed with the peptide and allowed to stand for 30 min at 25 °C. The reactions were then started by adding the buffer including Tris•HCl and MgCl_2_, so that each reaction contained 0.25 µM F1*/F1*sub, 50 mM Tris•HCl (pH 8.3), 25 mM MgCl_2_, 0.4% DMSO, and the peptides. The samples were incubated at 25 °C for 20 s, and then stopped by adding three volumes of stop buffer (8 M urea and 50 mM EDTA). After heat treatment (94 °C, 5 min), the samples were resolved on urea-PAGE gels (10–15% polyacrylamide, 8 M urea). The gels were analyzed using an Amersham Typhoon scanner.

### Activity test of the hammerhead ribozyme

The HH35 ribozyme was annealed with a labeled substrate, HPshortFAM, in 1 mM EDTA (pH 8.0), by heating the samples at 70 °C for 5 min and gradually decreasing the temperature to 12 °C. The ribozyme complex was mixed with the reaction buffer so that each reaction contained 0.25 µM HH35/HPshortFAM, 50 mM Tris•HCl (pH 8.3), 25 mM MgCl_2_, 0.4% DMSO, and the peptides. The samples were incubated at 25 °C for 20 s.

We also tested an experimental procedure in which the ribozyme and substrate were mixed in the presence of P43. We first annealed the HH35 ribozyme and its substrate, separately. The ribozyme and substrate were then mixed into the reaction buffer containing the peptide, and the samples were incubated at 17 °C for 3 min. The reactions were stopped by adding three volumes of stop buffer (8 M urea and 50 mM EDTA). After heat treatment (94 °C, 5 min), the samples were resolved on urea-PAGE gels (10–15% polyacrylamide, 8 M urea). The gels were analyzed using an Amersham Typhoon scanner.

### Microscopic observation of P43 aggregates

Aggregates of P43 were observed by an inverted microscope system (Nikon, Eclipse Ti, NIS-Elements AR 4.00.06 64-bit) under bright field illumination. FAM or FAM-labeled RNAs were added into a P43 suspension with MgCl_2_. These samples contained 4 mM P43 (2% DMSO), 20 µM FAM/FAM-RNAs, and 0–800 mM MgCl_2_. Aliquots (8 µL) of the P43/RNA complex suspensions were applied to cell counting slides (Bio-Rad) and visualized by using the fluorescence setup with the GFP filter set (excitation at 470 nm). The images were analyzed by using ImageJ (Java 1.8.0_172 64-bit).

### Negative-stain electron microscopy

Structures of P43 in the absence/presence of NTPs were observed by negative-stain electron microscopy (FEI, TF20). These samples contained 4 mM P43 (2% DMSO) / 800 µM P43 (0.4% DMSO), and 0/500 µM NTPs. Samples were applied to the carbon film grids and then stained with 2% uranyl acetate. EM images were obtained at a magnification of 50,000.

### Detection of peptide aggregates with ANS

To determine the critical aggregation concentrations (CACs) of peptides, 8-anilino-1-naphthalenesulfonic acid (ANS) was used as a fluorescent probe to detect aggregate formation. ANS is sensitive to the hydrophobic environment, making it suitable to detect the peptide aggregates with hydrophobic domains. The fluorescence spectra of 75 µM ANS with peptides were measured by a fluorescence spectrometer (JASCO, FP-8500DS, Spectra Manager Version 2 (2.14.05)) from 370 nm to 650 nm (λ_ex_ = 350 nm). The CACs were estimated by the fitting analysis of peptide concentration-dependent fluorescence intensity at 475 nm.

### Congo Red birefringence assays

Congo Red (CR) saturated solutions were prepared by dissolving powders of CR into 80% EtOH and removing the excess powder by filtration. The aggregated peptide suspensions were dried on slide glasses and stained by placing the CR solution onto the dried peptides for a few seconds, and then the excess CR solution was removed by blotting with a lab tissue. The imaging of stained samples was performed using polarized light microscopy.

### Statistics and reproducibility

For all ribozyme activity assays, each experiment was repeated at least three times independently in the same or similar conditions (Figs. 1f, 3a–c, 4b–g, 5b, c, 6b, d, f, and supplementary figures: three times; Fig. 1b: four times; Fig. 1d: five times). The peptide CAC measurements (Fig. 2a, bottom, Fig. 2b, Figs. S7b, S18, and S19) were repeated three times and the results were well reproduced except the CAC of P43 in the condition without NTP/MgCl_2_ (Fig. 2a, top) deviated largely when we used old samples stored in a freezer. Figure 2a shows the data with the freshly prepared sample. For Microscopic observations (fluorescent and electron microscopes), the experiments were repeated 3 times. All graphs show mean values ± SD.

### Reporting summary

Further information on research design is available in the Nature Research Reporting Summary linked to this article.

## Supplementary information

Supplementary Information

Reporting Summary

## Data Availability

The data supporting the findings of this study are available from the corresponding authors upon reasonable request. Source data for the figures and supplementary figures are provided as a Source Data file. Source data are provided with this paper.

## Source data

Source Data
