# Supplementary material for: Hydrophobic-cationic peptides modulate RNA polymerase ribozyme activity by accretion
Source: Nat Commun. 2022 Jun 3;13:3050. doi: 10.1038/s41467-022-30590-3 (PMC9166800; doi:10.1038/s41467-022-30590-3)
Supplement: Supplementary file 1 — Supplementary Information [file 41467_2022_30590_MOESM1_ESM.pdf]

Supporting Information

Hydrophobic-cationic peptides modulate RNA polymerase ribozyme activity by accretion

Peiying Li[1], Philipp Holliger[2*], Shunsuke Tagami[1,2*]

[1]*RIKEN Center for Biosystems Dynamics Research, 1-7-22 Suehiro-cho, Tsurumi-ku, Yokohama 230-0045, Japan.*

[2]*MRC Laboratory of Molecular Biology, Francis Crick Avenue, Cambridge Biomedical Campus, Cambridge CB2 0QH, UK.*

Corresponding Authors

* P.H. (ph1@mrc-lmb.cam.ac.uk), S.T. (shunsuke.tagami@riken.jp)

Supplementary Tables

Supplementary Table 1. DNA sequences

Application	Name	Manufacturer	Sequence
Ribozyme RNA polymerase	TC2	Japan Bio Services	TGTTCTGCCAACCGTGCGAAGCGTGGATTCATTG
F1* ribozyme assay	gF1*-F	Eurofins	CCTAATACGACTCACTATAGAGACCGCAACTGAAAAGTTGTTATCACTTG
	gF1*-C	Eurofins	CTGAAAAGTTGTTATCACTTGTCGTAAGACACTTTGGATGGGTTGAAGTTCT
	gF1*-R	Eurofins	GCCTTTTGCTTCTACGTGCAGAACTTCAACCCATCCAAAGTG

Supplementary Table 2. RNA sequences

Application	Name	Manufacturer	Sequence
Ribozyme RNA polymerase	Z^{LT}	IVT	GGACAACCAAAAAGACAAAUCUGCCCUCAGAGCUUGAGAACAUCUUCGGAUGCAGAGGAGGCAGCCUUCGGUGGCGCGAUAGCGCCAACGUUCUCAACAGACACCCAAUACUCCCGCUUCGGCGGGUGGGGAUAACACCUGACGAAAAGGCGAUGUUAGACACGCCCAGGUCAUAAUCCCCGGAGCUUCGGCUCCGCGGCCGCAAAAAAAAAAGGCUUACC
	Zt^{LT}	IVT	GGUCCGAAAGGACCAUCCACGCUUCGCACGGUUGGCAGAACAAACAAACAAACAAACAGGUUGUCCAGAUCUUCUUGAUCUGGACAACCAAAAAGACAAAUCUGCCCUCAGAGCUUGAGAACAUCUUCGGAUGCAGAGGAGGCAGCCUUCGGUGGCGCGAUAGCGCCAACGUUCUCAACAGACACCCAAUACUCCCGCUUCGGCGGGUGGGGAUAACACCUGACGAAAAGGCGAUGUUAGACACGCCCAGGUCAUAAUCCCCGGAGCUUCGGCUCCGCGGCCGCAAAAAAAAAAGGCUUACC
	FAM-R12 (RNA primer)	Japan Bio Services	FAM-CUGCCAACCGUG
	FAM-R11 (RNA primer)	Japan Bio Services	FAM-CUGCCAACCGU
	TI (RNA template)	Japan Bio Services	CAAUGAAUCCACGCUUCGCACGGUUGGCAGAACA
Phage display	Zc	IVT	GGAACAAAACACGCUGGCUAAUCAAAGACAAAUCUGCCCUCAGAGCUUGAGAACAUCUUCGGAUGCAGAGGAGGCAGCCUUCGGUGGCGCGAUAGCGCCAACGUUCUCAACAGACACCCAAUACUCCCGCUUCGGCGGGUGGGGAUAACACCUGACGAAAAGGCGAUGUUAGACACGCCCAGGUCAUAAUCCCCGGAGCUUCGGCUCC
	B1AP (RNA primer)	Sigma	GCCAGCG

RNase P assay	RNase P	IVT	GAAGCUGACCAGACAGUCGCCGCUUCGUCGUCGUCCUCUUCGGGGGAGACGGGCGGAGGGGAGGAAAGUCCGGGCUCCAUAGGGCAGGGUGCCAGGUAACGCCUGGGGGGGAAACCCACGACCAGUGCAACAGAGAGCAAACCGCCGAUGGCCCGCGCAAGCGGGAUCAGGUAAGGGUGAAAGGGUGCGGUAAGAGCGCACCGCGCGGCUGGUAACAGUCCGUGGCACGGUAAACUCCACCCGGAGCAAGGCCAAAUAGGGGUUCAUAAGGUACGGCCCGUACUGAACCCGGGUAGGCUGCUUGAGCCAGUGAGCGAUUGCUGGCCUAGAUGAAUGACUGUCCACGACAGAACCCGGCUUAUCGGUCAGUUUCACCU
	pATSerUG	Japan Bio Services	FAM-GAUCUGAAUGGAGAGAGGGGGUUCAAAUCCCCCUCUCUCCGCCAC
Hammerhead ribozyme assay	HH35 (ribozyme strand)	Japan Bio Services	GGGCAGCUGAUGAGUCCGUGAGGACGAAACUGUCA
	HPshortFAM (substrate strand)	Japan Bio Services	FAM-UGACAGUCCUGCCC
F1* ribozyme assay	F1* ligase (ribozyme strand)	IVT	GAGACCGCAACUGAAAAGUUGUUAUCACUUGUCGUAAGACACUUUGGAUGGGUUGAAGUUCUGCACGUAGAAGCAAAAGGC
	FAM-F1*sub	Japan Bio Services	FAM-GAGACCAAGAAACGUGCAGAAU
Microscopic observation	FAM-R1	Japan Bio Services	FAM-C
	FAM-R3	Japan Bio Services	FAM-CUG
	FAM-R5	Japan Bio Services	FAM-CUGCC
	FAM-R8	Japan Bio Services	FAM-CUGCCAAC
Precipitation assay	FAM-R11*2	Japan Bio Services	FAM-CUGCCAACCGUCUGCCAACCGU
	CFAM11	Japan Bio Services	ACGGUUGGCAG

Supplementary Figures

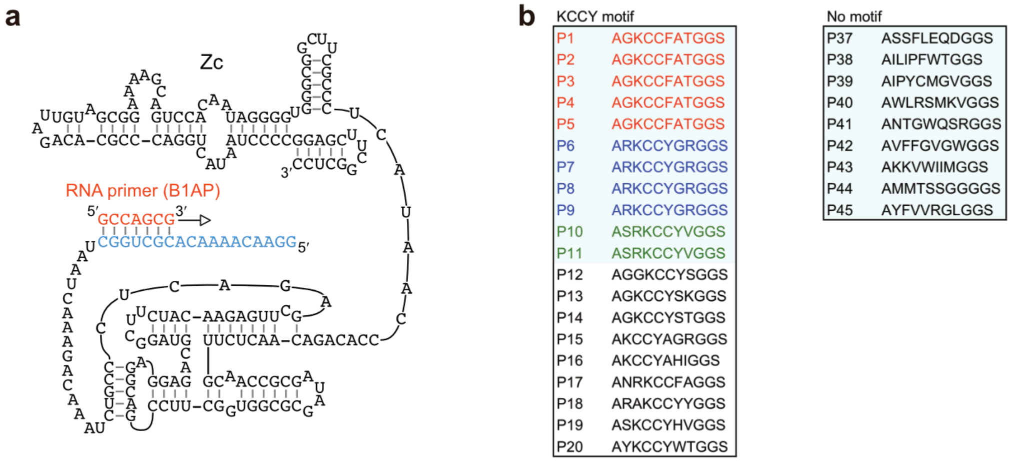

Supplementary Figure 1. The target RNA and selected peptides in the phage display. (a) Schematic depiction of the target RNA used in the phage display experiment (Zc). The 3′-biotinylated Zc ribozyme was annealed with the RNA primer (B1AP) in water (50ºC for 5 min, 17ºC for 10 min) before immobilization on the magnetic beads. (b) Sequences of the selected peptides. The results of rounds 3 and 4 are combined. Only sequences without any stop codons are shown.

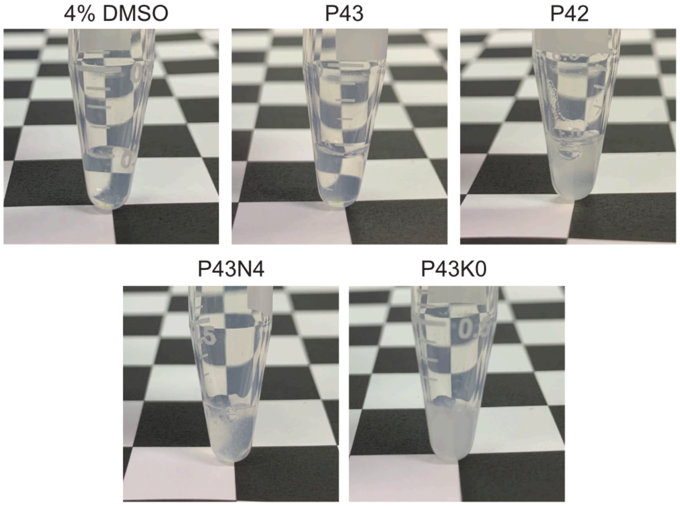

Supplementary Figure 2. Precipitates of the hydrophobic peptides. The tubes contained 2 mM peptides in water containing 4% DMSO. The P43 suspension is almost clear at this peptide concentration. P42, P43N4 and P43K0 showed large amounts of aggregates in the tubes, indicating that their solubilities were much lower than that of P43.

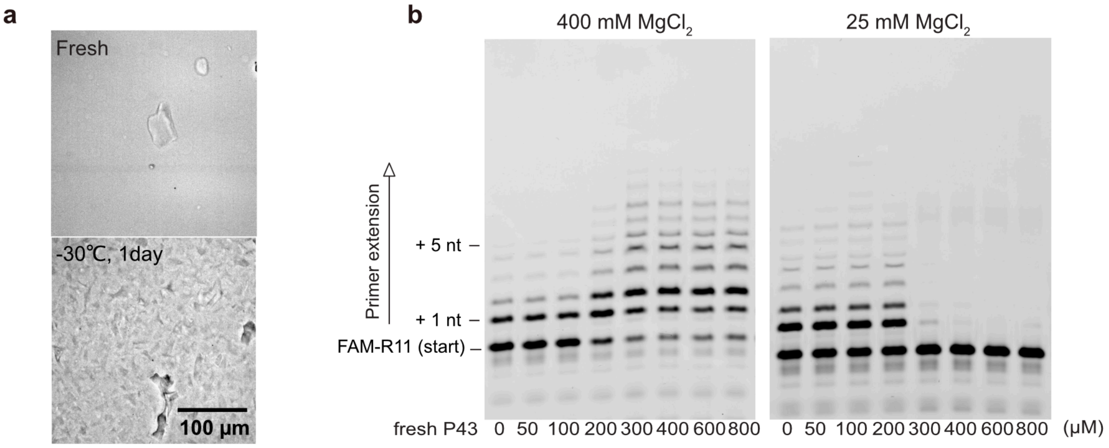

Supplementary Figure 3. Aggregate formation of the stored P43 sample. (a) The fresh 10 mM P43 (in 5% DMSO) stock suspension formed large aggregates after overnight storage in a −30ºC freezer. When the peptide was dissolved in DMSO and diluted with water, only a small amount of aggregates was detected in the suspension under bright field illumination (10 mM P43, 5% DMSO). However, after keeping the suspension in a freezer overnight or longer, a large amount of flake-like visible aggregates was formed. (b) The fresh P43 suspension was used in the RPR Z^{LT} reaction, and showed mostly the same effects on the RPR Z^{LT} as compared to the aged one in Figures 1c and 1d.

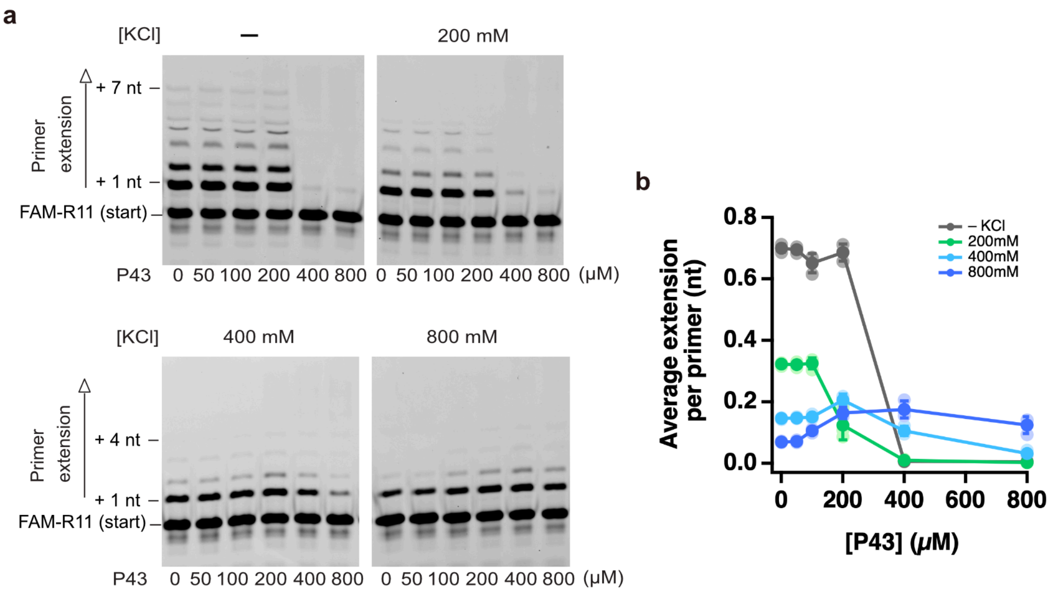

Supplementary Figure 4. The effect of the P43 peptide at high KCl concentrations. Primer extension by RPR Z^{LT} was performed in 50 mM Tris•HCl (pH 8.3) buffer containing 25 mM $MgCl_2$, 200–800 mM KCl, 8% PEG 6000, 0.4% DMSO and 500 µM of each NTP. The reactions were incubated at 17ºC for 7 days. Although the RPR activity was slightly inhibited at high KCl concentrations, the stimulative effect of P43 could still be observed as in the high $MgCl_2$ conditions. Data are presented as mean values ± S.D. (N = 3).

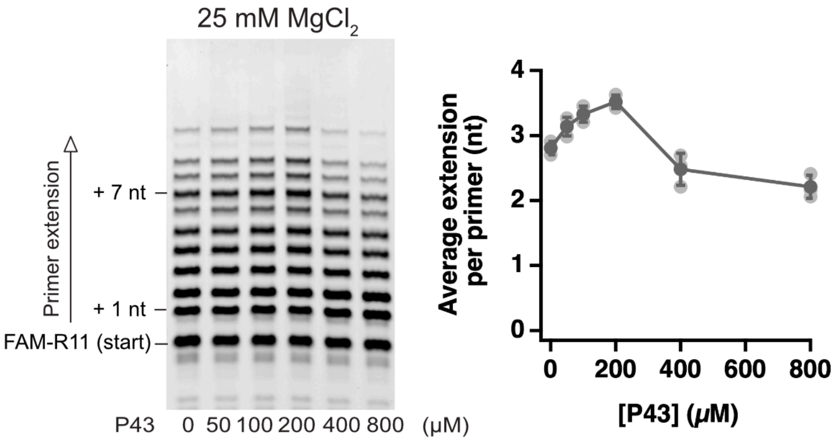

Supplementary Figure 5. The effect of the P43 peptide in eutectic ice phases. Primer extension by RPR Z^{LT} was performed in 50 mM Tris•HCl (pH 8.3) buffer containing 25 mM $MgCl_2$, 0.4% DMSO and 500 µM of each NTP. The reactions were incubated at −7ºC for 7 days after being frozen at −80ºC for 30 min. Data are presented as mean values ± S.D. (N = 3). In eutectic ice phases, the concentration of Mg^{2+} can be increased roughly four-fold, resulting in the higher activity of RPR.[1,2] Further stimulation of the RPR activity could be observed in the presence of 50–200 µM P43. However, the RPR activity was slightly decreased by 400–800 µM P43. The effect of P43 in the eutectic ice phase was more complicated than that in the non-frozen conditions (Figure 1), probably because not only [Mg^{2+}] but also all other materials (e.g., RNAs, P43, and NTPs) were concentrated.

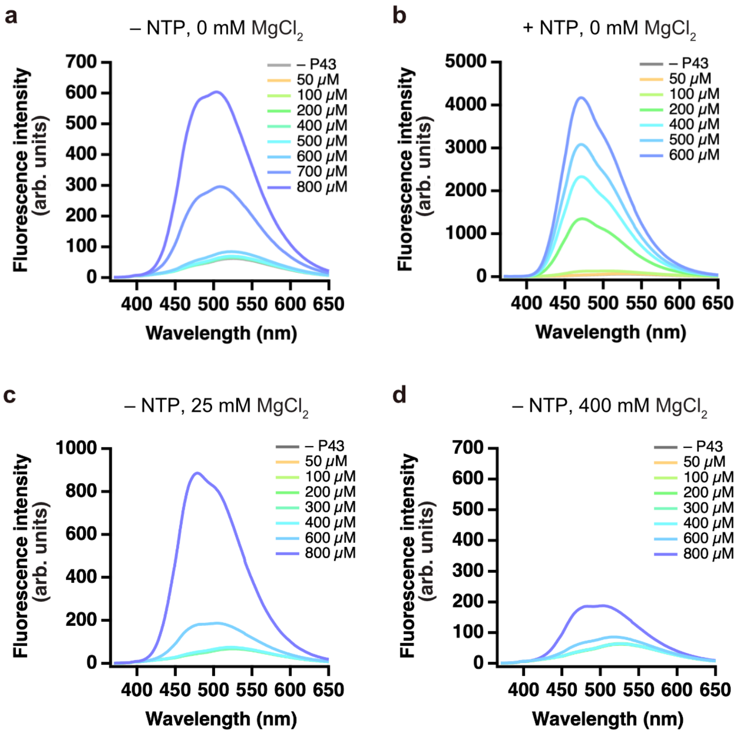

Supplementary Figure 6. Aggregation of P43 in water. Concentration-dependent aggregation of the P43 peptide was detected by the fluorescence intensity of ANS in water containing 0.4% DMSO (a), and in the presence of 500 µM of each NTP (b), or with 25 (c) or 400 (d) mM $MgCl_2$. In the absence of NTPs and $MgCl_2$, P43 formed aggregates from 600 µM, showing an increase of the fluorescence signal. With NTPs, P43 aggregates can be found even at 200 µM, indicating that NTPs can decrease the CAC of P43. In contrast, the $MgCl_2$ concentrations did not significantly affect the CACs of P43 in the absence of NTPs, which were around 500–600 µM P43.

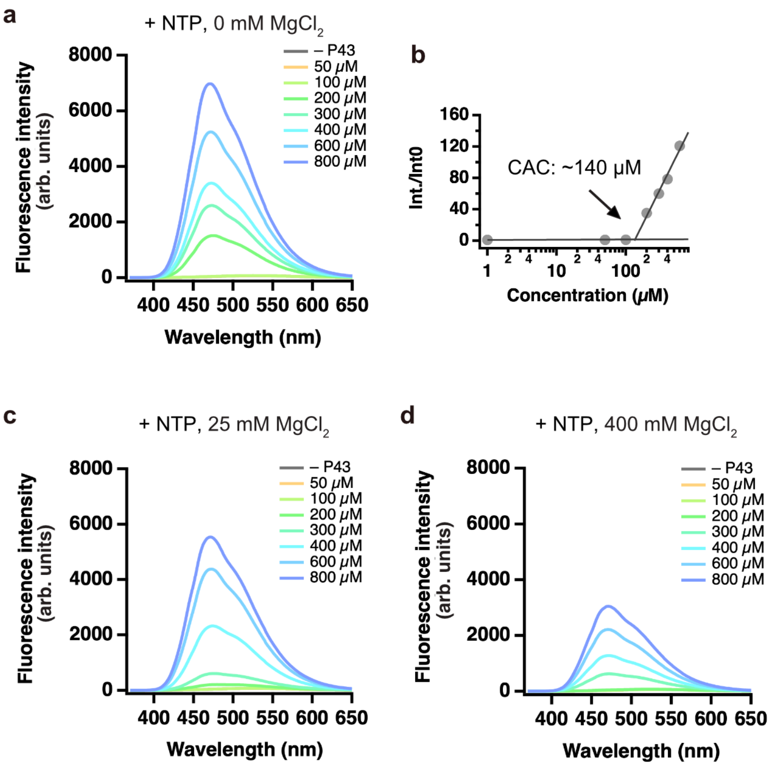

Supplementary Figure 7. Aggregation of P43 in the reaction buffer for RPR. Concentration-dependent aggregation of the P43 peptide was detected by the fluorescence intensity of ANS in the reaction buffer for RPR (50 mM Tris•HCl (pH 8.3), 0.4% DMSO, and 500 μM of each NTP) with 0 (a), 25 (c) or 400 (d) mM $MgCl_2$. (b) The CAC of P43 at 0 mM $MgCl_2$ was evaluated by the fitting analysis of the concentration-dependent fluorescence intensity at 475 nm (a). The estimated CAC is almost the same in water and the reaction buffer for RPR (also see Figure 2a and Supplementary Figure 6b). (c, d) In the presence of 25 or 400 mM $MgCl_2$, the CAC of P43 became slightly higher (200–300 μM) with $MgCl_2$.

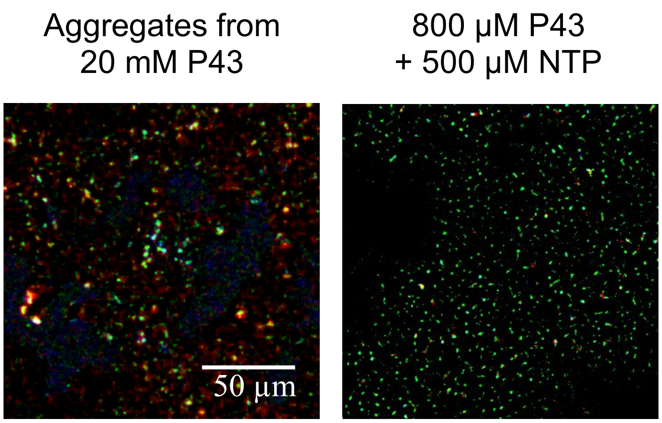

Supplementary Figure 8. The P43 aggregates stained with Congo Red. (Left) The aggregates in the P43 stock suspension (20 mM P43 in 10% DMSO) were precipitated by centrifugation, gently washed with H_2O for 4 times to remove the DMSO, and then dispersed in H_2O. This suspension was dried on a glass slide and stained by Congo Red. (Right) Aggregates of 800 μM P43 with 500 μM of each NTP were prepared by simply mixing the samples, drying them on glass slides, and staining by Congo Red.

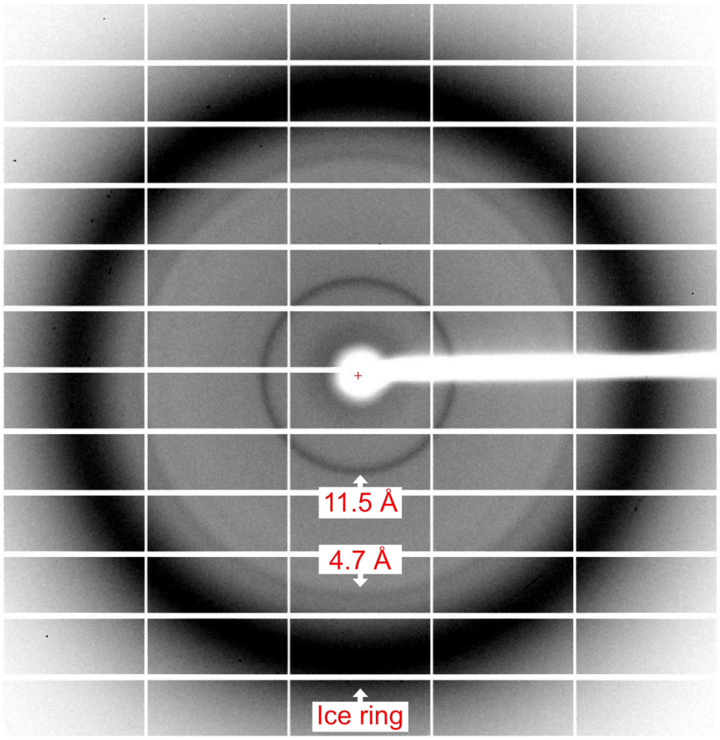

Supplementary Figure 9. X-ray diffraction data from the P43 aggregates. The peptide aggregates in 10 mg/mL P43 in 10% DMSO were precipitated by centrifugation, captured with a protein crystallography loop, and frozen with liquid N_2. The X-ray diffraction data were obtained by synchrotron radiation (KEK, PF BL5A). Diffractions at 4.7 Å and ~10 Å are general features of cross-β amyloid,[3] indicating that the P43 aggregates from the high concentration sample at least partially adopt the cross-β conformation. Because the P43 aggregates were not aligned, the diffraction pattern was not anisotropic.

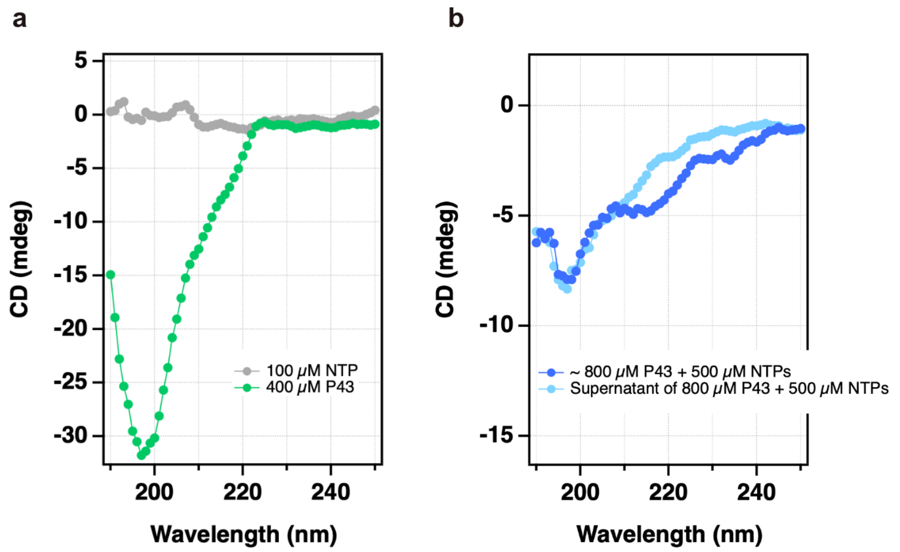

c

Sample	A_{260}	A_{280}	A_{260}/A_{280}
800 µM P43	2.61 ± 0.01	4.39 ± 0.03	0.59
500 µM NTPs	22.52 ± 0.27	11.04 ± 0.16	2.04
Supernatant of 800 µM P43 + 500 µM NTPs	20.38 ± 0.13	10.58 ± 0.21	1.93

Supplementary Figure 10. CD spectra of the P43 peptide. (a, b) The aggregates in the P43 stock suspension (20 mM P43 in 10% DMSO) were precipitated by centrifugation and gently washed 8 times with H_2O without disrupting the precipitates to remove the DMSO, which prevents measurements of the CD spectra. We then estimated the amounts of the remaining precipitates by measuring the peptide concentrations of the supernatant and washes, and adjusted the sample concentrations for the CD measurements by adding H_2O to the precipitates. (a) The P43 sample without NTP was adjusted to 400 µM, a concentration lower than its CAC (Figure 2a), and remained well dissolved. (b) The P43 sample with NTPs was adjusted to the concentration used in the RPR reaction as a suspension (800 µM P43, 500 µM NTPs). The aggregates in the mixture were precipitated by centrifugation and gently washed twice with H_2O

to remove the excess NTPs. We then dispersed the aggregates into H_2O to form a suspension again. For the CD spectra measurement, 1 mm-pathlength cuvettes were filled with 200 μL of samples. The CD spectra were scanned from 250 nm to 190 nm at 20ºC, using a JASCO J820 CD spectrometer. The 400 μM P43 solution without NTPs (a) showed the typical CD spectra of random coiled peptides with a negative peak at 198 nm (green). In contrast, the suspension of 800 μM P43 with NTPs exhibited another negative peak at 215 nm (b, blue). When we centrifuged this sample again and measured the CD spectra of the supernatant, it showed only the negative peak at 198 nm (b, cyan), which superimposed well on the 198 nm peak from the suspension. Thus, the random coil feature of the CD spectra of the suspension was from dissolved peptides, while the peak at 215 nm was from the aggregates with β structures in the sample. However, we could not estimate the ratio between random coil and β structures in the suspension because aggregates larger than the wavelength mostly scatter instead of absorbing light, which would result in CD signals strongly biased to smaller aggregates and solubilized peptides. CD spectra of 100 μM NTPs are also shown (a, gray). (c) Absorbances at 260 nm and 280 nm of 800 μM P43, 500 μM NTPs, and the supernatant of their mixture were measured using a NanoDrop (Thermo Scientific). The mixture of 800 μM P43 and 500 μM NTPs was prepared by combining 4 mM P43, 5 mM NTPs, and H_2O and centrifuging it to obtain its supernatant. As the A_{260} of the supernatant is ~90% of 500 μM NTPs, only ~10% of mixed NTPs (50 μM) was estimated to be co-precipitated with P43.

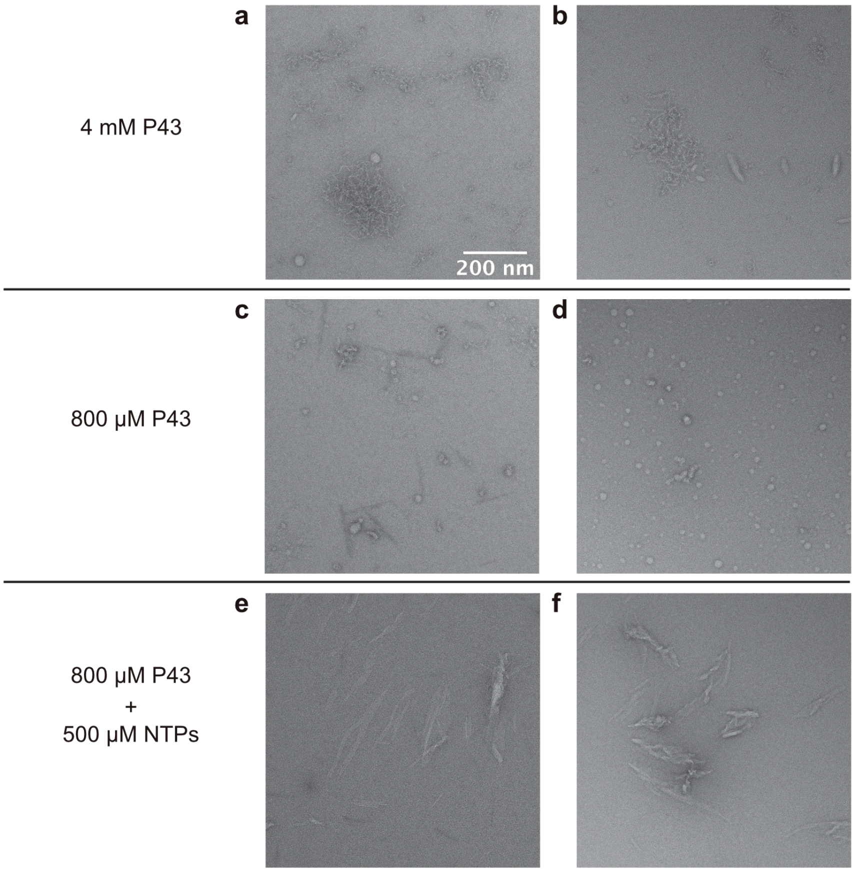

Supplementary Figure 11. Electron microscopy analysis of the P43 aggregates. The P43 aggregates were observed by negative-stain electron microscopy. (a, b) 4 mM P43 in 2% DMSO. (c, d) The P43 aggregates at the concentration (800 µM P43 in 0.4% DMSO) used in the activity test of the ribozymes. (e, f) The aggregates of 800 µM P43 with 500 µM NTPs in 0.4% DMSO. While the sample without NTPs contained aggregates in various forms (lumpy and elongated structures), the sample with NTPs had larger bundled forms. The NTPs might have led to the flocculation of the elongated aggregates via a charge complementarity that reduces the repulsion of the positively-charged peptide molecules.

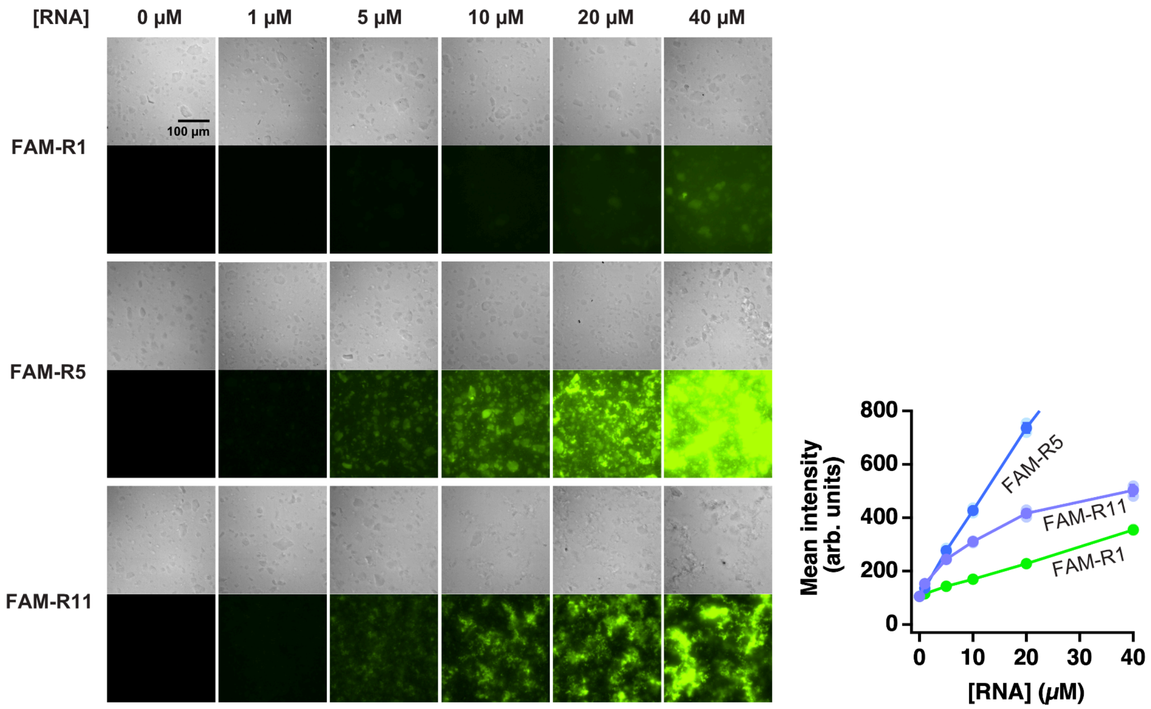

Supplementary Figure 12. P43 aggregates bound with RNAs. (Left) Microscopic analysis of fluorescently-labeled RNAs bound to the P43 aggregates. The peptide aggregates (4 mM, 2% DMSO) were mixed with fluorescently-labeled RNAs (FAM-R1, FAM-R5, and FAM-R11) at various concentrations (0, 1, 5, 10, 20, and 40 μM), and observed with bright-field and fluorescence-filter setups (FAM, exposure time = 30 ms). (Right) The average fluorescence intensities of the RNAs on the peptide aggregates. Data are presented as mean values ± S.D. (N = 3).

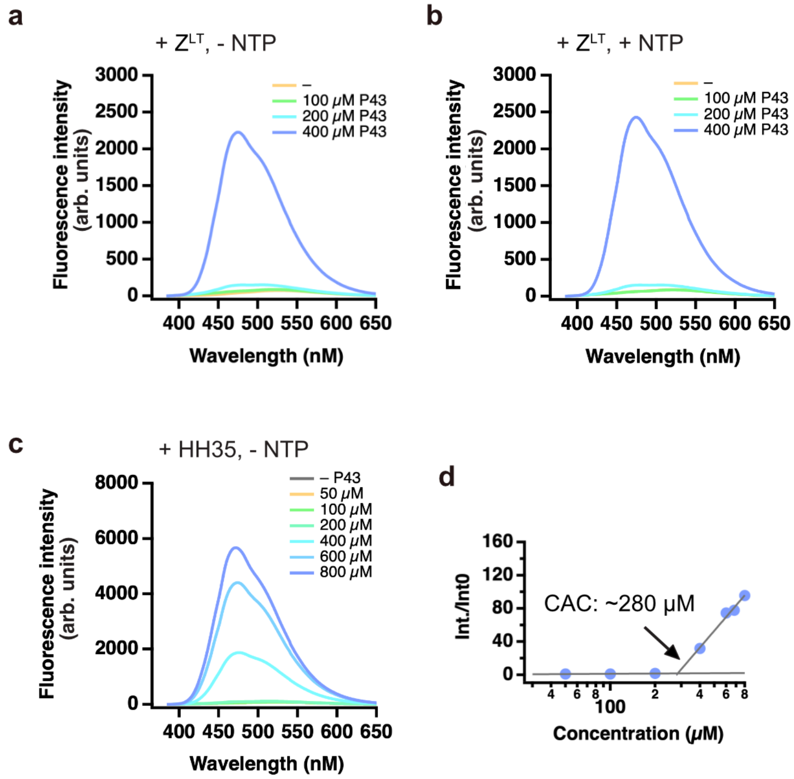

Supplementary Figure 13. Aggregation of P43 with RNA. (a, b) Fluorescence intensities of ANS with 0, 100, 200, and 400 µM P43 and 0.25 µM Z^{LT} were measured in 50 mM Tris•HCl (pH 8.3) buffer containing 25 mM $MgCl_2$ and 0.4% DMSO, in the absence (a) or presence (b) of 500 µM of each NTP. (c) Fluorescence intensities of ANS were measured in the presence of 0–800 µM P43 and 0.25 µM HH35 in 50 mM Tris•HCl (pH 8.3) buffer containing 25 mM $MgCl_2$ and 0.4% DMSO. (d) The CAC of P43 with HH35 was evaluated by the fitting analysis of the peptide concentration-dependent fluorescence intensity at 475 nm (c). These results demonstrated that small amounts of RNAs can stimulate the formation of P43 aggregates even without NTPs.

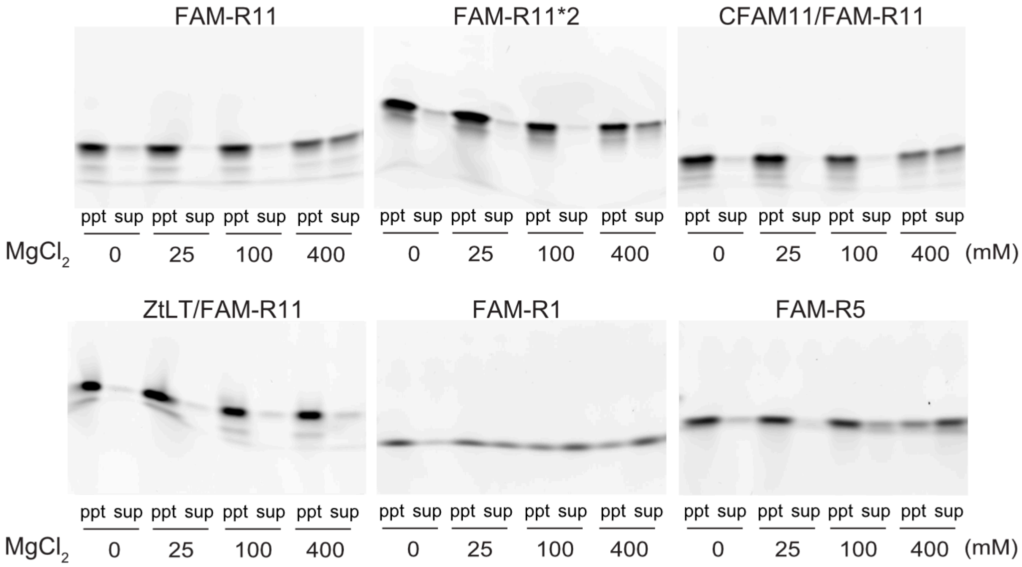

Supplementary Figure 14. Co-precipitation assays of P43 and RNAs. The P43 suspensions (800 μM, 0.4% DMSO) were mixed with fluorescently-labeled RNAs (0.25 μM), in 50 mM Tris•HCl (pH 8.3) buffer containing 0, 25, 100, and 400 mM $MgCl_2$, and were then divided into precipitates and supernatants by centrifugation. The precipitated fractions of all tested RNAs decreased as the $MgCl_2$ concentration increased, indicating that the large amounts of $MgCl_2$ weakened the interactions between P43 and RNA. We also found that the size of RNAs has a large effect on the binding affinity (Figure 2e).

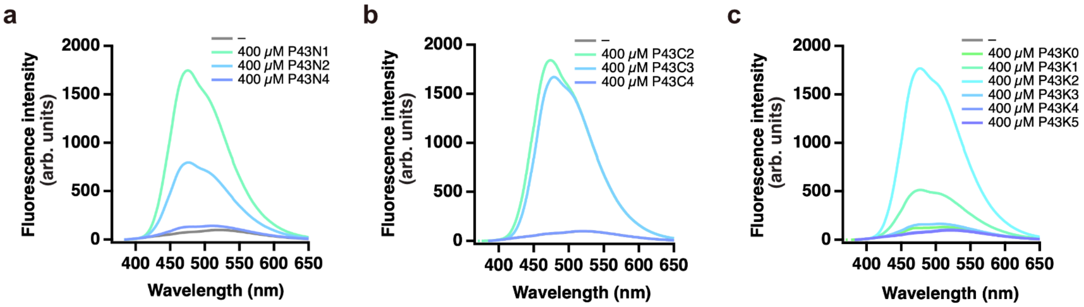

Supplementary Figure 15. Aggregation of P43 variants. (a-c) Fluorescence intensity of ANS in a 400 µM solution/suspension of P43 variants containing 50 mM Tris•HCl (pH 8.3), 25 mM $MgCl_2$, 0.8% DMSO and 500 µM of each NTP. P43N1, P43C2, P43C3, P43K2, and P43K3 formed aggregates with NTPs, which showed strong inhibitory effects on the activity of RPR (Figure 3a, b). Even though P43N2, P43N4, P43K0, and P43K1 also formed aggregates, probably due to the weaker interactions with RPR, the inhibitory effects were not significant. P43K4 and P43K5 were soluble even in the presence of NTPs.

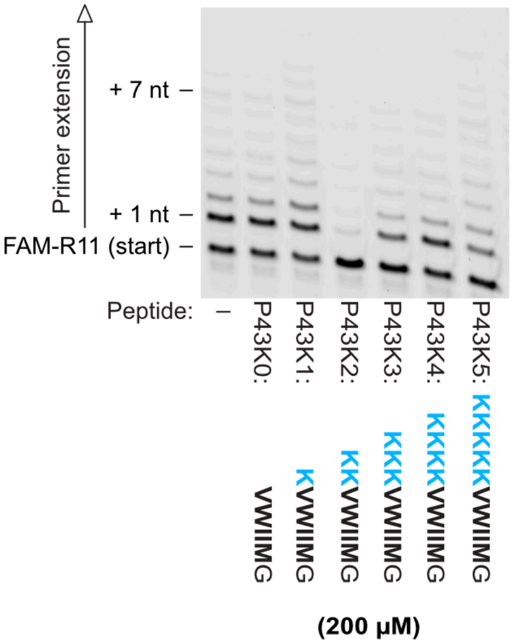

Supplementary Figure 16. P43 variants with different numbers of lysine residues. Primer extension by RPR Z^{LT} was performed in 50 mM Tris•HCl (pH 8.3) buffer containing 25 mM $MgCl_2$, 8% PEG 6000, 0.4% DMSO and 500 µM of each NTP with 200 µM P43 variants. The reactions were incubated at 17ºC for 7 days. Only P43K2 showed a significant inhibitory effect.

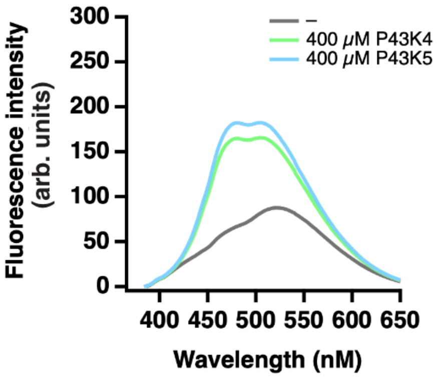

Supplementary Figure 17. Aggregation of P43 variants with RPR Z^{LT}. Fluorescence intensities of ANS in 400 μM P43K4 and P43K5 with 0.25 μM Z^{LT} were measured in 50 mM Tris•HCl (pH 8.3) buffer containing 25 mM $MgCl_2$, 0.8% DMSO, and 500 μM of each NTP. Although P43K4 and P43K5 were soluble even with NTPs (Supplementary Figure 15c), adding RPR Z^{LT} accelerated the aggregate formation.

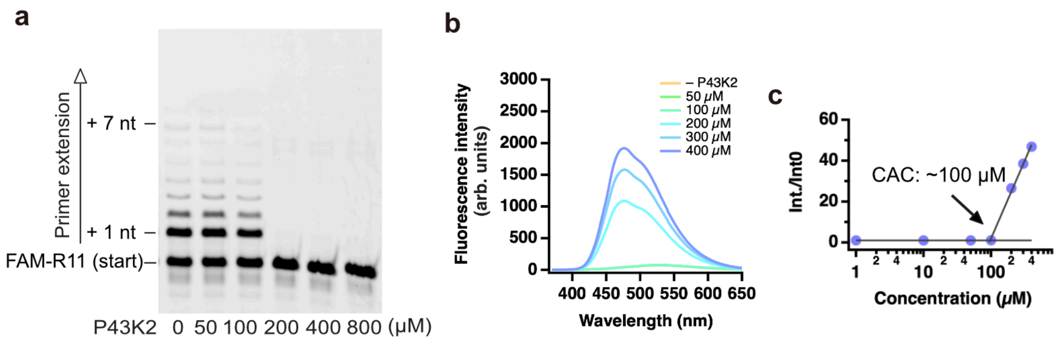

Supplementary Figure 18. Aggregation of P43K2. (a) Effect of P43K2 on the activity of RPR Z^{LT}. Primer extension by RPR Z^{LT} was performed in 50 mM Tris•HCl (pH 8.3) buffer containing 25 mM $MgCl_2$, 8% PEG 6000, 0.4% DMSO and 500 µM of each NTP. The reactions were incubated at 17ºC for 7 days. (b) Fluorescence intensities of ANS with P43K2 were measured in 50 mM Tris•HCl (pH 8.3) buffer containing 25 mM $MgCl_2$, 0.4% DMSO, and 500 µM NTPs. (c) The CAC of P43K2 was estimated by the fitting analysis of the peptide concentration-dependent fluorescence intensity at 475 nm (b). As compared to P43, P43K2 showed a lower CAC, demonstrating that the non-essential A, G, and S residues of P43 still affect the aggregate formation. P43K2 also showed a strong inhibitory effect at 200 µM (also see Supplementary Figure 16), while 200 µM P43 did not inhibit RPR (Figure 1c, d). These results indicate that the inhibitory effects of P43 variants are correlated to their CACs.

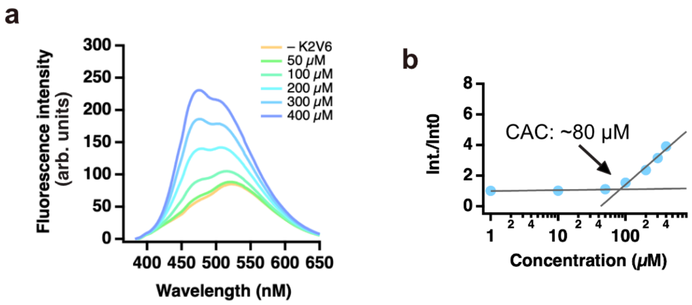

Supplementary Figure 19. Aggregation of K_2V_6. (a) Fluorescence intensity of ANS with K_2V_6 was measured in 50 mM Tris•HCl (pH 8.3) buffer containing 25 mM $MgCl_2$, 0.4% DMSO, and 500 µM NTPs. (b) The CAC of K_2V_6 was estimated by the fitting analysis of the peptide concentration-dependent fluorescence intensity at 475 nm (a). K_2V_6 showed a lower CAC (~80 µM) than P43 under the same conditions (~260 µM, Figure 2b).

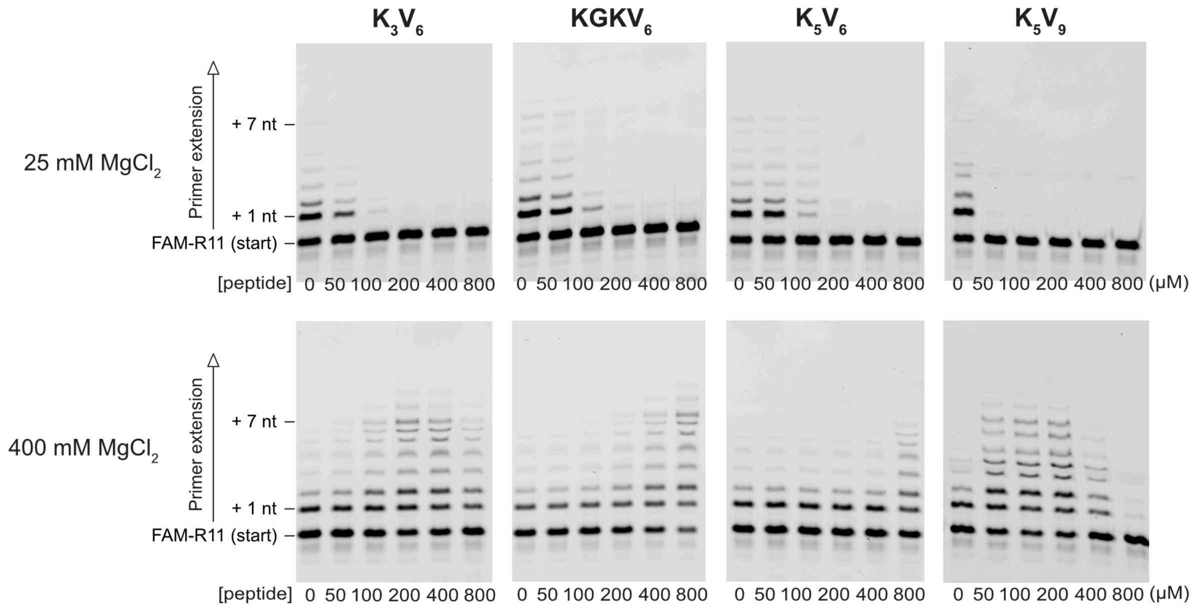

Supplementary Figure 20. Effects of various hydrophobic-hydrophilic peptides. Primer extension by RPR Z^{LT} was performed in 50 mM Tris•HCl (pH 8.3) buffer containing 25 mM or 400 mM $MgCl_2$, 8% (25 mM $MgCl_2$) or 0% (400 mM $MgCl_2$) PEG 6000, 0.4% DMSO and 500 µM of each NTP. The reactions were incubated at 17ºC for 7 days (25 mM $MgCl_2$) or 3 days (400 mM $MgCl_2$).

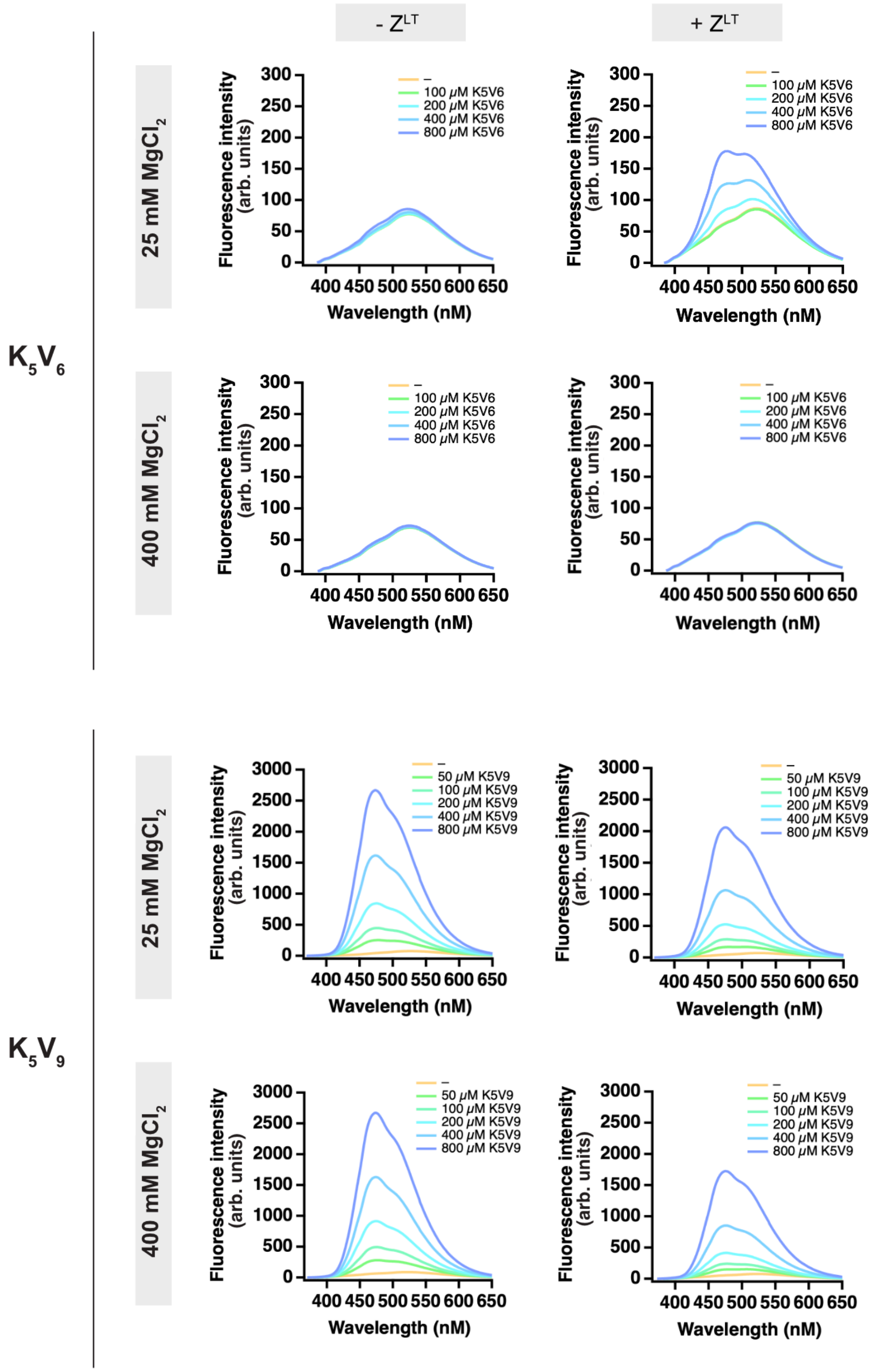
- Z^LT
+ Z^LT
K5V6
K5V9
25 mM MgCl2
400 mM MgCl2
Fluorescence intensity (arb. units)
Wavelength (nM)
–
50 μM K5V9
100 μM K5V9
200 μM K5V9
400 μM K5V9
800 μM K5V9
100 μM K5V6
200 μM K5V6
400 μM K5V6
800 μM K5V6

Supplementary Figure 21. Aggregation of K_5V_6 and K_5V_9 with RPR Z^{LT}. Fluorescence intensity of ANS. The K_5V_6 and K_5V_9 peptides were measured in the absence or presence of Z^{LT} in 50 mM Tris•HCl (pH 8.3) buffer containing 25 mM or 400 mM $MgCl_2$, 0.4% DMSO and 500 µM of each NTP. K_5V_6 formed aggregates only with Z^{LT} at the low $MgCl_2$ concentration. The high $MgCl_2$ concentration inhibited the aggregate formation, indicating that electrostatic interactions between K_5V_6 and RNA also contributed to the aggregation of the peptide. In contrast, the peptide with more valine residues, K_5V_9, formed aggregates in all tested conditions.

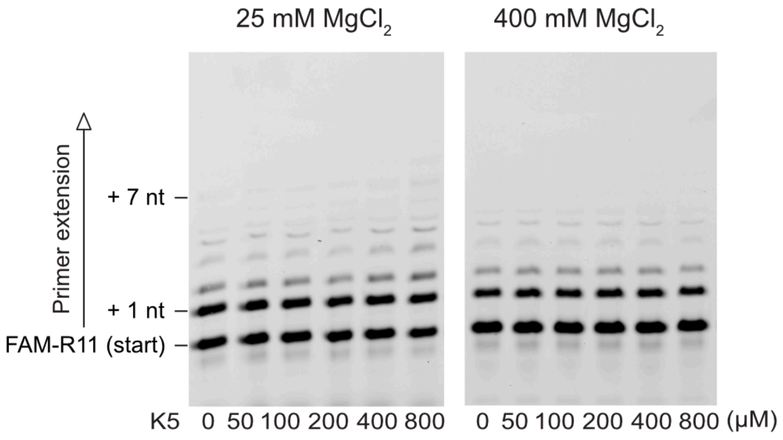

Supplementary Figure 22. Effect of K_5 on the activity of RPR Z^{LT}. Primer extension by RPR Z^{LT} was performed in 50 mM Tris•HCl (pH 8.3) buffer containing 25 mM or 400 mM $MgCl_2$, 8% (25 mM $MgCl_2$) or 0% (400 mM $MgCl_2$) PEG 6000, 0.4% DMSO and 500 µM of each NTP. The reactions were incubated at 17ºC for 7 days (25 mM $MgCl_2$) or 3 days (400 mM $MgCl_2$). K_5 did not show significant effects at both low and high $MgCl_2$ concentrations.

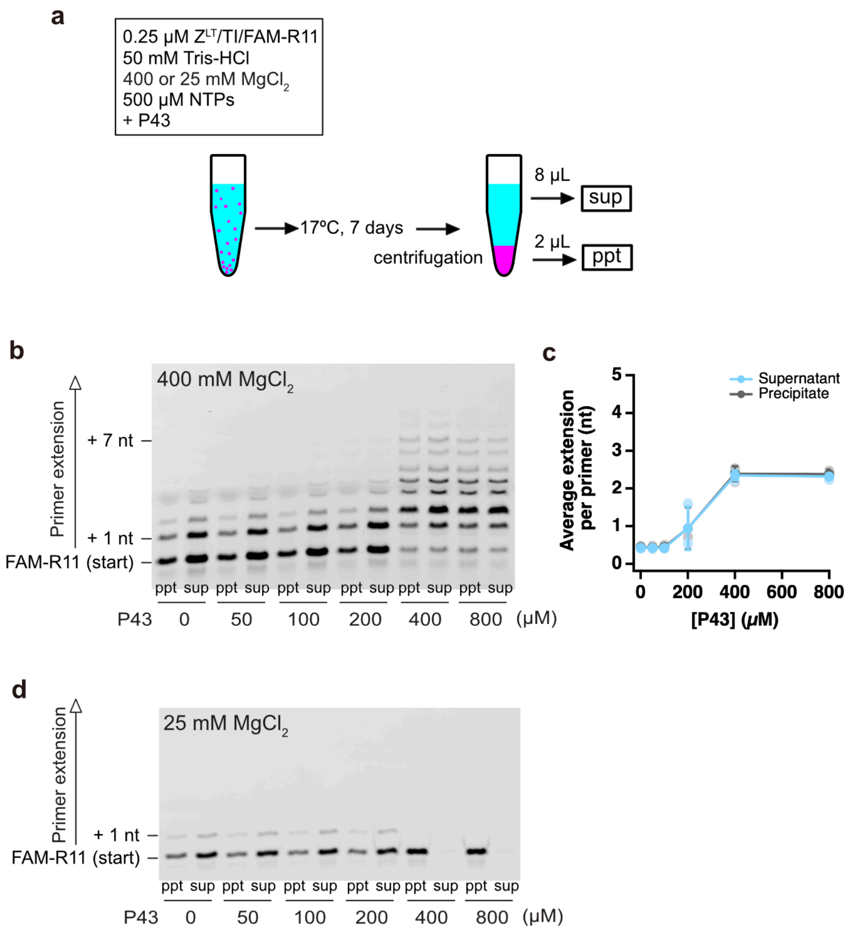

Supplementary Figure 23. Separation into precipitates and supernatants after the RPR reaction. (a) Schematic depiction of the experimental procedure. (b–d) Primer extension by RPR Z^{LT} was performed in 50 mM Tris•HCl (pH 8.3) buffer containing 400 mM (b, c) or 25 mM (d) $MgCl_2$, 0.4% DMSO and 500 μM of each NTP. The RPR reactions were divided into supernatant and precipitate after a 3 (b, c) or 7 (d) -day incubation at 17ºC. At 400 mM $MgCl_2$, there was no difference between the average primer extensions in the two fractions. At 25 mM $MgCl_2$, most of the RNA primer precipitated when the P43 concentration was higher than its CAC (≥ 400 μM), showing no primer extension by RPR. (c) Data are presented as mean values ± S.D. (N = 3).

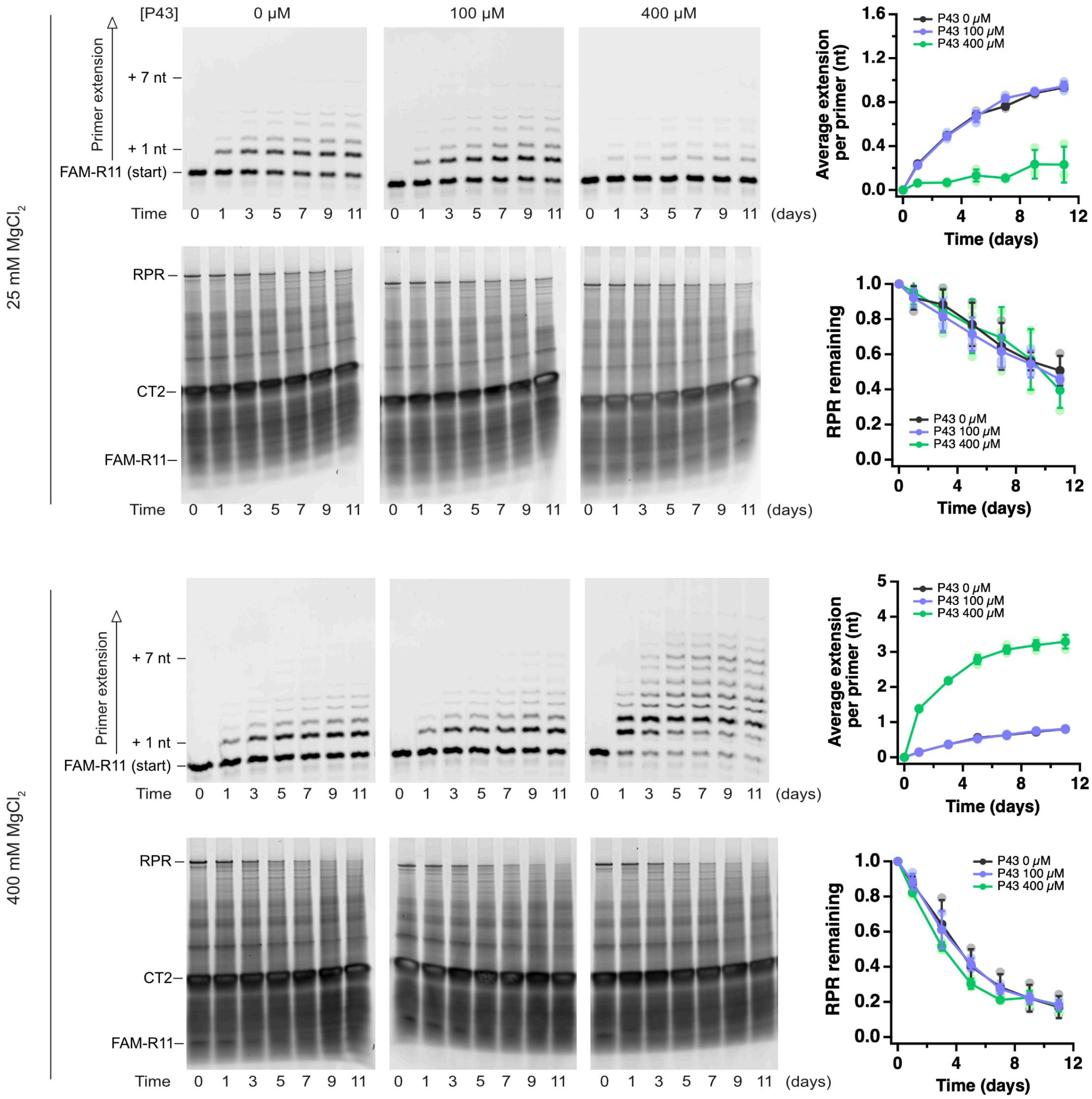

Supplementary Figure 24. Degradation of RPR in the presence of P43. Time-course analysis of Z^{LT} activity and degradation. Primer extension by RPR Z^{LT} was performed in 50 mM Tris•HCl (pH 8.3) buffer containing 25 mM or 400 mM $MgCl_2$, 8% (25 mM $MgCl_2$) or 0% (400 mM $MgCl_2$) PEG 6000, 0.4% DMSO and 500 µM of each NTP. The reactions were incubated at 17ºC for 0–11 days. The integrity of RPR after the incubation was analyzed by staining the gel with SYBR Gold. Z^{LT} degraded at almost the same rates in the absence and presence of P43. Data are presented as mean values ± S.D. (N = 3).

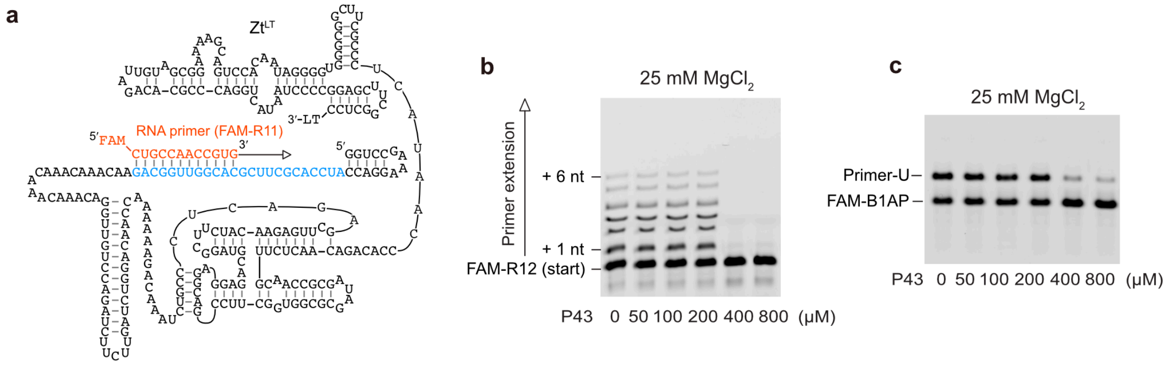

Supplementary Figure 25. P43 effects on different RPR constructs. (a) Schematic depiction of Zt^{LT}. 3′-LT is a 3′-extension sequence (GCGGCCGCAAAAAAAAAAGGCUUACC) used in the previous ribozyme selection for RPR Z.[4] (b) Primer extension by RPR Zt^{LT} was performed in 50 mM Tris•HCl (pH 8.3) buffer containing 25 mM $MgCl_2$, 0.4% DMSO and 500 µM of each NTP. The reactions were incubated at 17°C for 60 min. (c) Primer extension by RPR Zc was performed in 50 mM Tris•HCl (pH 8.3) buffer containing 25 mM $MgCl_2$, 0.4% DMSO and 2 mM of UTP. The reactions were incubated at 17°C for 10 min. P43 at 400–800 µM showed inhibitory effects on the activities of both Zt^{LT} and Zc at the low [Mg^{2+}], similar to the case with Z^{LT}.

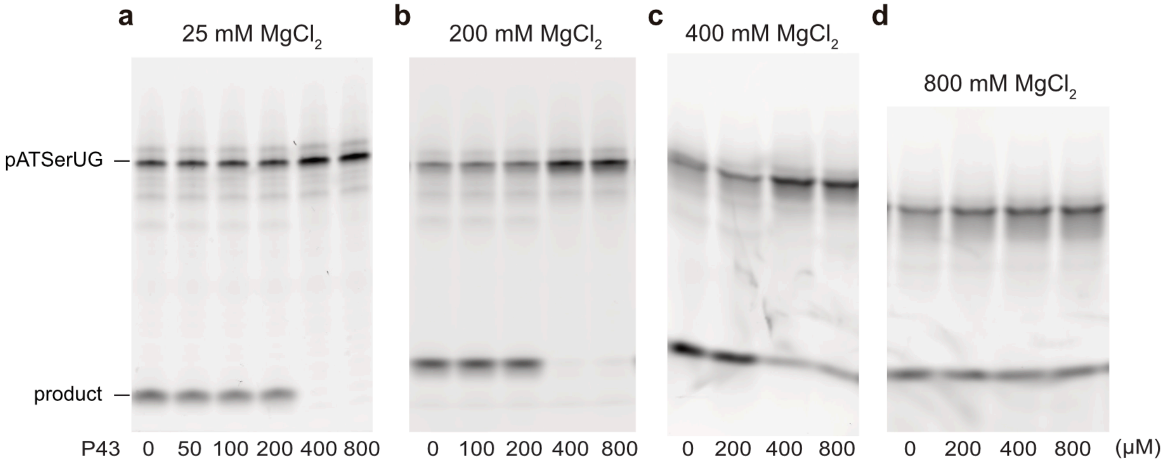

Supplementary Figure 26. Inhibition of the RNase P activity by the P43 peptide. (a–d) The RNase P activity was tested in the presence of the P43 peptide. The reactions were performed in (a) 50 mM Tris•HCl (pH 8.0) buffer containing 25 mM $MgCl_2$ and 0.4% DMSO at 37ºC for 10 min, (b–d) 50 mM Tris•HCl (pH 8.0) buffer containing 200–800 mM $MgCl_2$ and 0.4% DMSO at 37ºC for 2.5 min.

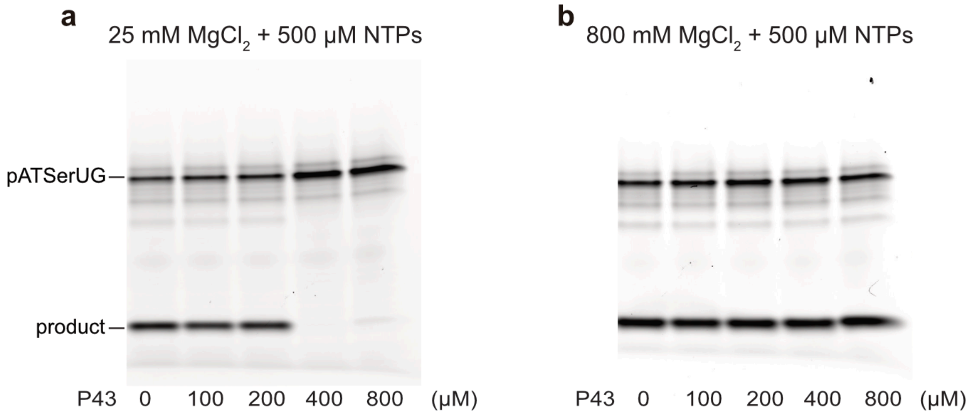

Supplementary Figure 27. Inhibition of the RNase P activity by the P43 peptide in the presence of NTPs. (a, b) The RNase P activity was tested in the presence of the P43 peptide and NTPs. The reactions were performed in (a) 50 mM Tris•HCl (pH 8.0) buffer containing 25 mM $MgCl_2$, 0.4% DMSO and 500 µM NTPs at 37ºC for 10 min, (b) 50 mM Tris•HCl (pH 8.0) buffer containing 800 mM $MgCl_2$, 0.4% DMSO and 500 µM NTPs at 37ºC for 2.5 min.

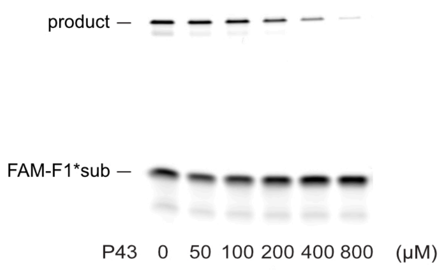

Supplementary Figure 28. Inhibition of the F1* activity by the P43 peptide. The F1* activity was tested in the presence of the P43 peptide. The reactions were performed in 50 mM Tris•HCl (pH 8.3) buffer containing 25 mM $MgCl_2$ and 0.4% DMSO at 25ºC for 20 sec.

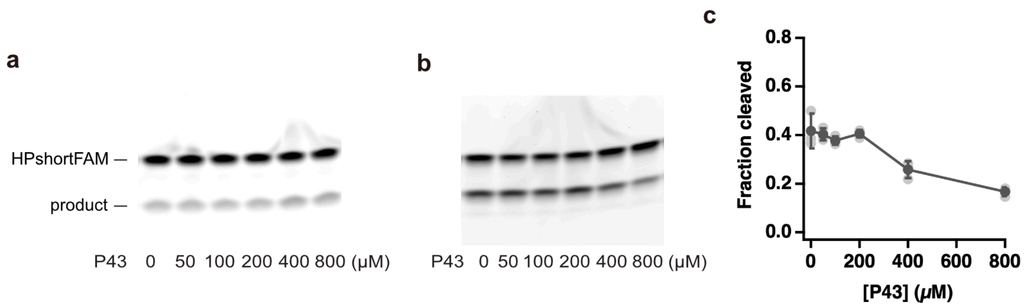

Supplementary Figure 29. The HH35 activity is affected by the P43 peptide. (a) HH35 was first annealed with the substrate in water. The RNA cleavage reaction by HH35 was then performed in 50 mM Tris•HCl (pH 8.3) buffer containing 25 mM $MgCl_2$ and 0.4% DMSO, in the presence of P43, at 25ºC for 20 sec. (b, c) HH35 and the substrate were annealed separately in water. The ribozyme and substrate were then sequentially mixed into the reaction buffer containing the peptide. The reaction was performed in 50 mM Tris•HCl (pH 8.3) buffer containing 25 mM $MgCl_2$ and 0.4% DMSO, at 25ºC for 3 min. Data are presented as mean values ± S.D. (N = 3).

References

(1) Attwater, J., Wochner, A., Pinheiro, V. B., Coulson, A., and Holliger, P. (2010) Ice as a protocellular medium for RNA replication. *Nat. Commun. 1*, 76.

(2) Attwater, J., Wochner, A., and Holliger, P. (2013) In-ice evolution of RNA polymerase ribozyme activity. *Nat. Chem. 5*, 1011–1018.

(3) Nelson, R., and Eisenberg, D. (2006) Structural Models of Amyloid-Like Fibrils, in *Advances in Protein Chemistry. 73*, pp 235–282.

(4) Wochner, A., Attwater, J., Coulson, A., and Holliger, P. (2011) Ribozyme-Catalyzed Transcription of an Active Ribozyme. *Science (80-.). 332*, 209–212.
