# Supplementary material for: Hydrophobic-cationic peptides modulate RNA polymerase ribozyme activity by accretion
Source: Nat Commun. 2022 Jun 3;13:3050. doi: 10.1038/s41467-022-30590-3 (PMC9166800; doi:10.1038/s41467-022-30590-3)
Supplement: Supplementary file 2 — Reporting Summary [file 41467_2022_30590_MOESM2_ESM.pdf]

nature portfolio

Corresponding author(s): Shunsuke Tagami

Last updated by author(s): Apr 27, 2022

Reporting Summary

Nature Portfolio wishes to improve the reproducibility of the work that we publish. This form provides structure for consistency and transparency in reporting. For further information on Nature Portfolio policies, see our Editorial Policies and the Editorial Policy Checklist.

Statistics

For all statistical analyses, confirm that the following items are present in the figure legend, table legend, main text, or Methods section.

n/a	Confirmed	
☐	☒	The exact sample size (*n*) for each experimental group/condition, given as a discrete number and unit of measurement
☐	☒	A statement on whether measurements were taken from distinct samples or whether the same sample was measured repeatedly
☒	☐	The statistical test(s) used AND whether they are one- or two-sided<br>*Only common tests should be described solely by name; describe more complex techniques in the Methods section.*
☒	☐	A description of all covariates tested
☒	☐	A description of any assumptions or corrections, such as tests of normality and adjustment for multiple comparisons
☐	☒	A full description of the statistical parameters including central tendency (e.g. means) or other basic estimates (e.g. regression coefficient) AND variation (e.g. standard deviation) or associated estimates of uncertainty (e.g. confidence intervals)
☒	☐	For null hypothesis testing, the test statistic (e.g. *F*, *t*, *r*) with confidence intervals, effect sizes, degrees of freedom and *P* value noted<br>*Give P values as exact values whenever suitable.*
☒	☐	For Bayesian analysis, information on the choice of priors and Markov chain Monte Carlo settings
☒	☐	For hierarchical and complex designs, identification of the appropriate level for tests and full reporting of outcomes
☒	☐	Estimates of effect sizes (e.g. Cohen's *d*, Pearson's *r*), indicating how they were calculated

Our web collection on statistics for biologists contains articles on many of the points above.

Software and code

Policy information about availability of computer code

Data collection	Amersham Typhoon 1.1.0.7 was used for the scan of urea-PAGE gels.<br><br>NIS-Elements AR 4.00.06 64-bit was used to image the peptide aggregates under bright field illumination and the fluorescence of FAM-labeled RNAs.<br><br>Spectra Manager Version 2 (2.14.05) was used to detect and record the fluorescence spectra of ANS in peptide samples.<br><br>Spectra Manager for Windows 95/NT (1.51.00) was used to detect and record the CD spectra of peptide samples.
Data analysis	ImageQuant TL 1D version 8.1 was used to analyze the urea-PAGE gels.<br><br>ImageJ 1.52k (Java 1.8.0_172 64-bit) was used to analyze the fluorescence images of FAM-labeled RNAs.<br><br>Igor Pro 8 (64-bit) was used to create graphs and estimate the CACs by the fitting analysis of peptide concentration-dependent fluorescence intensity.

For manuscripts utilizing custom algorithms or software that are central to the research but not yet described in published literature, software must be made available to editors and reviewers. We strongly encourage code deposition in a community repository (e.g. GitHub). See the Nature Portfolio guidelines for submitting code & software for further information.

Data

Policy information about availability of data

All manuscripts must include a data availability statement. This statement should provide the following information, where applicable:

- Accession codes, unique identifiers, or web links for publicly available datasets
- A description of any restrictions on data availability
- For clinical datasets or third party data, please ensure that the statement adheres to our policy

The data that support the findings of this study are available from the corresponding authors only on reasonable request as some gels contain unpublished data.

Field-specific reporting

Please select the one below that is the best fit for your research. If you are not sure, read the appropriate sections before making your selection.

☒ Life sciences ☐ Behavioural & social sciences ☐ Ecological, evolutionary & environmental sciences

For a reference copy of the document with all sections, see nature.com/documents/nr-reporting-summary-flat.pdf

Life sciences study design

All studies must disclose on these points even when the disclosure is negative.

Sample size	Minimal repeat number of enzymatic analysis was three. We determined this to be sufficient as the enzymatic activity was calculated with internal control (ratio between the products and substrates in the same samples). For the conditions in which the enzymatic activity was relatively unstable, we repeated the experiments five times (Fig 1d).
Data exclusions	No data were excluded.
Replication	Samples for data replication were separately prepared. Quantifications were performed using the same programs and equations applied to all conditions and replications. All replication attempts were successful, except the CAC measurement of P43 without NTP/Mg (Figure 2a, top) largely deviated when we used old samples. The data reported in Figure 2a was obtained with a newly-bought freshly-prepared sample.
Randomization	We used highly purified molecules and no biological samples (like cells or animals). Thus, randomization was not relevant to our experiments.
Blinding	Blinding was not possible as biochemical experimental conditions were evident. Quantifications were performed using the same programs and equations applied to all conditions and replications.

Reporting for specific materials, systems and methods

We require information from authors about some types of materials, experimental systems and methods used in many studies. Here, indicate whether each material, system or method listed is relevant to your study. If you are not sure if a list item applies to your research, read the appropriate section before selecting a response.

Materials & experimental systems

n/a	Involved in the study	
☒	☐	Antibodies
☒	☐	Eukaryotic cell lines
☒	☐	Palaeontology and archaeology
☒	☐	Animals and other organisms
☒	☐	Human research participants
☒	☐	Clinical data
☒	☐	Dual use research of concern

Methods

n/a	Involved in the study	
☒	☐	ChIP-seq
☒	☐	Flow cytometry
☒	☐	MRI-based neuroimaging
